# Digital range of motion analysis is sensitive to subjective steps in joint model construction

**DOI:** 10.1111/joa.70179

**Published:** 2026-06-03

**Authors:** R. J. Lowes, A. Jannel, B. W. Griffin, T. L. Prescott, P. L. Falkingham

**Affiliations:** ^1^ School of Biological and Environmental Sciences Liverpool John Moores University Liverpool UK

**Keywords:** avian, biomechanical modelling, joint mobility, range of motion, six degrees of freedom

## Abstract

Reconstructing locomotion and behaviour in extinct vertebrates requires a detailed understanding of joint mobility to constrain the range of potential limb orientations to more biologically plausible poses. Joint mobility is typically assessed using range of motion (ROM) analysis, which is increasingly implemented within digital workflows. Digital ROM analysis requires the positioning of bones in three‐dimensional space into a starting pose that enables systematic sampling of rotational and translational configurations (e.g. reference pose). However, this initial process involves subjective steps, particularly in selecting articular surfaces and defining a joint centre of rotation. No thorough sensitivity analysis has yet been published. In this study, we have conducted systematic sensitivity analyses of a complete six degree‐of‐freedom automated ROM analysis workflow to evaluate how variation at each stage of the reference pose assembly, stemming from both input data and user decision, affects the determined viable poses in the ankle and tarsometatarsophalangeal III joints of Guinea fowl (*Numida meleagris*). Our results reveal that ROM analysis outputs are sensitive to variation in reference pose assembly, especially changes in articular surface selection and the primitive used to define the joint centre. This sensitivity may be unlikely to directly affect the overall conclusions of any given individual study, particularly when using maximum viable rotations to constrain biomechanical models. However, it potentially makes comparison between studies and taxa problematic, and we therefore advocate that future ROM studies should prioritise providing complete joint models as supplemental data to enable replicability.

## Graphical abstract

A systematic sensitivity analysis of a six degree‐of‐freedom range of motion (ROM) analysis workflow was conducted to evaluate how variation in the subjective steps of the reference pose assembly affects the determined viable poses in the ankle and tarsometatarsophalangeal III joints of Guinea fowl (*Numida meleagris*). Our results reveal that ROM analysis outputs are sensitive to this variation, especially changes in articular surface selection and the primitive used to define joint centre.

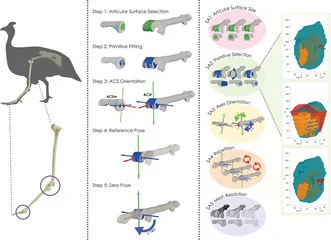

## 1 INTRODUCTION

Understanding how extinct animals move provides key insights into their evolutionary adaptations, ecology and behaviour. As direct in vivo observation is not possible in fossils, biomechanical modelling is necessary to provide insight into the motion of extinct taxa (Bishop et al., 2021). Reconstructing locomotion and behaviour in extinct vertebrates requires a detailed understanding of joint mobility to constrain the range of potential limb orientations to more biologically plausible poses (Gatesy et al., 2009).

Joint mobility can be assessed through range of motion (ROM) analysis. In extant animals, including humans, ROM analysis can involve goniometry (Hancock et al., 2018; Jaeger et al., 2007), dissections with mechanical testing (Herbst et al., 2022; Wilke et al., 2017), sensor‐based systems (Chiang et al., 2017; Costa et al., 2020), and 2D or 3D digital motion analysis (Anderst et al., 2008; Herbst et al., 2022; Kambic et al., 2014; Kim et al., 2015; Peebles et al., 2021). In extinct animals, ROM analysis has traditionally involved manually rotating physical, and later virtual, bones until either direct bone‐to‐bone collision occurs or a visually defined disarticulation is reached, the latter being dependent on the experimenter's judgement. This is deemed the osteological or “bone‐on‐bone” method (Mallison, 2010a, 2010b; Senter & Robins, 2005; Senter & Sullivan, 2019; White et al., 2015). Typically, this method employs three degrees of freedom (3DOF), with bones rotated around X, Y and Z‐axes (usually corresponding to long‐axis rotation (LAR), abduction/adduction (ABAD) and flexion/extension (FE)) without translations (Manafzadeh & Gatesy, 2022). Minimum and maximum angles of each axis are often measured independently without inter‐axis consideration; however, in vivo data have revealed that substantial interaction between degrees of freedom occurs (Kambic et al., 2017).

Digitally, this ROM approach has been adapted to enable automated techniques for exhaustive sampling of rotations that detect collisions and identify non‐interpenetrating viable poses. Manafzadeh and Padian (2018) introduced an automated exhaustive pose sampling method, using Boolean intersection operations for interpenetration detection. This has subsequently been extended to include all six degrees of freedom (6DOF) (Manafzadeh & Gatesy, 2021). 6DOF ROM analysis results in a more accurate representation of true joint mobility, as it removes the assumption of a fixed centre of rotation by enabling translational movements (Bishop et al., 2022; Demuth et al., 2025; Manafzadeh & Gatesy, 2021). It has been demonstrated that many in vivo joint poses are missed when only rotational degrees of freedom are allowed (Manafzadeh & Gatesy, 2021; Scheidt et al., 2026). This 6DOF approach allows all poses to be judged for viability, hence why it has been adopted by many later studies (Brocklehurst et al., 2022; Demuth et al., 2020; Griffin et al., 2022; Jones et al., 2021; Manafzadeh & Gatesy, 2021; Regnault & Pierce, 2018). Other automated techniques have been developed, including APSE (Accelerated Pose Searching with Electrostatics), which generally has less processing time due to identifying viable poses using an expanding search space algorithm, rather than sampling every possible configuration sequentially (Bishop et al., 2022).

Digital ROM analysis requires rearticulation of the digital bone models comprising a joint within a virtual environment (Gatesy et al., 2022; Manafzadeh & Gatesy, 2021). To do so, a centre of rotation for each joint needs to be identified. Joint centres are often determined by fitting geometric primitives to the articular surfaces, either manually fitting by eye (Brocklehurst et al., 2022, 2025; Costa et al., 2014; Fahn‐Lai et al., 2018; Molnar et al., 2021; Pierce et al., 2012) or selecting the vertices that make up the articular surface and fitting automatically (Bishop et al., 2021; Demuth et al., 2020, 2025; Gatesy et al., 2022; Richards et al., 2021). Whilst both these steps involve subjectivity, and many authors acknowledge the potential sensitivity in the reference pose assembly (Bishop et al., 2021; Gatesy et al., 2022; Manafzadeh & Gatesy, 2021), no thorough sensitivity analysis has yet been published. Preliminary sensitivity analyses have revealed changes in maximum joint angles when changing primitive shape or moving fixed joint centre position (Demuth et al., 2020; Díez Díaz et al., 2025; Richards et al., 2021), however, none have systematically quantified these effects. Digital 6DOF ROM analysis also relies on a subjective or arbitrary determination of disarticulation to constrain the included translations. To mitigate this, Jones et al. (2021) suggested prohibiting certain distances between anatomical landmarks to maintain articulation, recreating the maximum possible soft tissue strain. Richards et al. (2021), proposed using another mesh formed by extruding along the vertex normals of one articular surface to represent the disarticulation threshold. In the APSE approach, articular surface overlap and separation distance thresholds can be used as a measure of disarticulation, although this relies on subjective values for each threshold (Bishop et al., 2022). Whilst these methods mitigate some subjectivity, they are still dependent on assumptions about maximum joint spacing, which necessitates in situ data.

Each method relies on the assembly of a “neutral” or “reference” pose, from which all rotations occur (Bishop et al., 2021; Manafzadeh & Gatesy, 2021). This process was made as objective and repeatable as possible by Gatesy et al. (2022), who proposed a standard approach for reference pose assembly of archosaur hindlimbs. However, it still involves subjective processes, particularly the manual selection of the articular surfaces which determine the position of the primitives. Whilst the results of many of these methods seem plausible, thorough sensitivity analyses should be performed to test subjective model assumptions in all biomechanical analyses (Hicks et al., 2015). In this study, we have conducted systematic sensitivity analyses of the complete 6DOF automated method to assess how variation in each stage of the reference pose assembly affects the determined viable poses. We assessed the sensitivity in various aspects of the assembly process by changing the manual selections by the user, the input data, and the general workflow. Understanding how these changes impact the viable poses and the overall sensitivity involved in ROM analysis can help inform interpretation of ROM analysis results and guide future advancements.

## 2 MATERIALS AND METHODS

### 2.1 Data acquisition

*Numida meleagris* (helmeted guinea fowl) ankle and tarsometatarsophalangeal III (TMTP3) joints were chosen for the simplicity of uniaxial hinge joints. CT data were obtained from a prior study (Falkingham & Gatesy, 2014), which was re‐segmented using Dragonfly 3D World (Comet Technologies Canada Inc., 2025). The segmentation resulted in triangulated bone meshes of the right tibiotarsus, right tarsometatarsus, and right proximal phalanx of digit III (hereafter referred to as “phalanx III‐1”). Each model included 204,418 faces, 111,530 faces, and 26,172 faces, respectively.

### 2.2 Reference/zero pose assembly

The bone meshes were then assembled into reference poses in Blender 5.0 (Blender Online Community, 2025), following the protocol proposed by Gatesy et al. (2022) (Figure 1). This involves manually selecting the vertices that make up the articular surfaces, then fitting cylinders to the distal articular surfaces and planes to the proximal articular surfaces. Shapes were fit using the Blender Shape Fitter add‐on (https://github.com/pfalkingham/blenderShapeFitter). Whilst the ankle joint set up matches Gatesy et al. (2022), their study did not directly discuss standards for the phalanges; however, it can be assumed that the same protocol should be followed for TMTP3. The centres of these geometric primitives determine the centre of rotation of each articular surface, where anatomical coordinate systems (ACS) were placed using the XROMM Blender Tools extension (https://github.com/pfalkingham/XROMM_BlenderTools), each consisting of three orthogonal axes (XYZ). The ACS fitted to the distal primitive (ACSf) remains fixed in space during joint rotations, and the ACS fitted to the proximal primitive (ACSm) is mobile. The bone meshes were parented to their respective ACSm, then each ACSm was positioned at the same location and orientation as the corresponding ACSf and parented to create the reference pose. The ACSf of TMTP3 was rotated 180° around the Z‐axis so the phalanx III‐1 was directed straight out, then the rotations were reset to 0° to create the zero pose. All rotations and translations occur from this zero pose, rather than the reference pose, to more intuitively represent the plantarflexion and dorsiflexion that occurs at TMTP3 and to match previous work on this joint (Manafzadeh et al., 2024). The ankle remained in the reference pose and all rotations occurred from here, where 0°, 0°, 0° represents a fully flexed joint with no ABAD or LAR.

**FIGURE 1:**
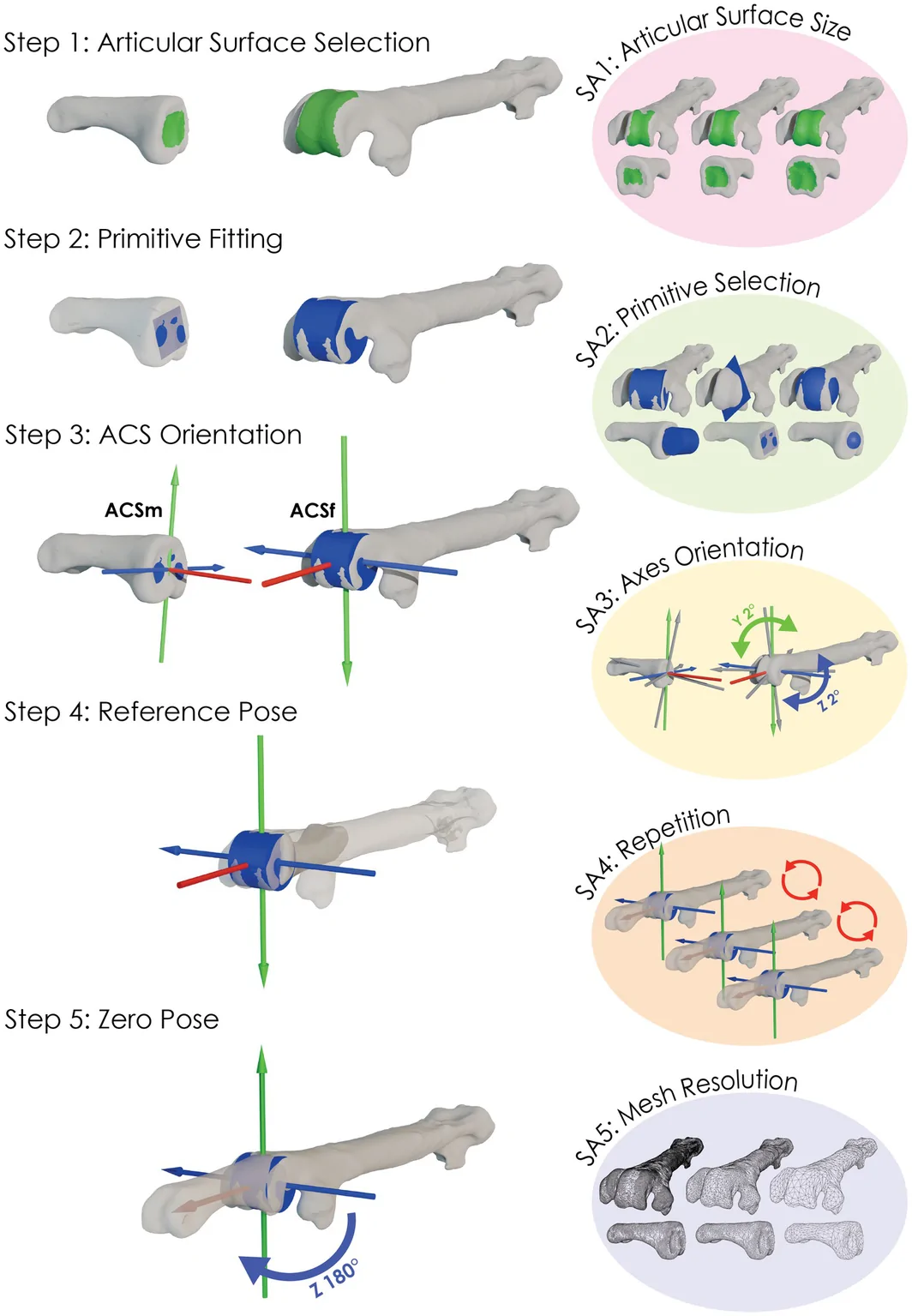
Reference pose assembly process of the TMTP3 joint using *Numida meleagris* (helmeted guinea fowl) tarsometatarsus and phalanx III‐1 bone models, generally following the standard process proposed by Gatesy et al. (2022). X‐, Y‐ and Z‐axes are red, green and blue, respectively. Sensitivity analyses performed are shown in ovals down the right‐hand side. Axes orientations for SA3 are for illustration purposes only and do not reflect the actual axes orientations tested. The same assembly process and sensitivity analyses was performed for the ankle joint using tibiotarsus and tarsometatarsus bone meshes, except the creation of a zero pose in step 5.

### 2.3 Sensitivity analysis

Sensitivity analyses were conducted to assess how variation at each stage of the reference or zero pose assembly affected the overall workflow and ROM analysis outcomes (Figure 1). The selection of the articular surfaces is one of the most subjective steps in reference pose assembly, and the size of the selection could vary between trials and between researchers; therefore, a sensitivity analysis was performed to test how changes in the articular surface selection size affect ROM analysis outcomes (SA1).

Gatesy et al. (2022) recommend using cylinders for distal articular surfaces and planes for proximal articular surfaces, but there may be cases where a researcher feels this shape is not appropriate for their particular bone. As a result, different primitive combinations may affect the determined joint centre of rotation and the resulting viable poses. To test this, different primitive types were fitted to each articular surface (SA2).

Joint axes orientation is influenced by the primitives fitted to the other end of the bones that are not directly involved in the joint, as the X‐axes within the joint are directed towards these primitives. To test the sensitivity of the results to variation in this step, the joint axes were manually rotated to systematically simulate the change in location of the indirect primitives (SA3).

To assess the repeatability of the reference pose assembly, each articular surface was reselected multiple times whilst keeping all other articular surface selections the same. This was to see whether similar articular surfaces could be selected consistently, and how small variations in model construction affect results (SA4).

Finally, multiple mesh resolutions were tested. Multiple aspects of selection and collision are determined by vertex and face density and location. We therefore resampled meshes to determine if lower resolution bone meshes affect model construction and subsequent ROM outputs (SA5).

#### 2.3.1 SA1: Changing size of articular surface selection

The articular surfaces were first selected based on the suggestions of Gatesy et al. 2022. Using the same base selections each time, the “select more” or “select less” tools in Blender were then applied once and twice for each articular surface involved in the target joint. The “select more” tool expands the vertex selection to all adjacent vertices, whereas the “select less” tool reduces the selection by removing the outermost vertices. Standard primitives per Gatesy et al. (2022) were then fitted to each articular surface alternative and the remaining reference pose assembly process was followed (as described in 2.1). Translation ranges depended on the parameters of the cylinder fitted to the distal tarsometatarsus for the TMTP3 and the distal tibiotarsus for the ankle (see 2.4); therefore, varied between trials. This resulted in a total of 25 trials for each joint.

#### 2.3.2 SA2: Changing primitive

Cylinder, plane and sphere primitives were fitted to the bone models using the same articular surface selection for each, resulting in 9 trials per joint. When fitting sphere and plane primitives to the distal articular surfaces on each bone, ACSf orientation differed substantially from what is to be expected of ACSs. This is because the ACSf Z‐axis is determined by the orientation of the primitive's axes, which are not aligned correctly with the bone unlike when using a cylinder. To account for this, axes were manually rotated to expected positions and rotations were recorded for ease of repeatability (Table S1). This was not necessary for each ACSm, as when directing the X‐axis to the distal primitive, the Z‐axis followed the direction of the distal primitive's Z‐axis. No manual translation of the axes was performed, regardless of whether the centre looked positioned correctly or not, as the aim of changing primitives is to test how different shapes alter the centre of rotation using the automated process. Translation ranges were kept the same for all trials, based on the values determined by the cylinders fitted to the distal articular surface of the proximal bone for repeatability purposes (see 2.4), although the actual 3D joint space caused by the X‐translation depended on the position of the ACSm.

#### 2.3.3 SA3: Changing axes orientation

When fitting the axes to the centre of the primitives, the ACSf and ACSm within each joint were manually rotated by ±2° around either the Y‐ or Z‐axis. The remaining reference pose assembly process (as described in 2.1) was then completed for each alternate orientation. This resulted in a total of 25 trials per joint. As the X‐axes of the ACSs are targeted towards the primitives on the other end of the bones that are not involved in the joint, changes in ACSf orientation simulate different positioning of the plane fitted to the proximal articular surface of the proximal bone within the joint, and changes in ACSm represent different positioning of the cylinder fitted to the distal articular surface of the distal bone. These rotations likely exaggerate the degree of variation in primitive fitting, as large differences in the placement of these primitives only result in very small differences in the orientation of the axes involved in the joint, but were considered appropriate for testing the sensitivity of the parameter. Changing the axes orientation manually allowed for more systematic testing, rather than attempting to alter the primitive centroid positions by changing the articular surface selections.

#### 2.3.4 SA4: Repetition of articular surface selection

Ten different reference poses were generated for each joint by RJL following the same protocol using the same bone meshes (Gatesy et al., 2022), changing only either the proximal or distal articular surface selection each time. First, only the proximal bone's distal articular surface was changed, creating five reference poses and aiming to select the same articular surface each time. This determined cylinder size and placement, which also affected translation ranges. All other articular surfaces and primitives were kept consistent. Then the same process was repeated with only changing the distal bone's proximal articular surface selection, creating another five reference poses. This meant that the plane position and proportions changed, but translation ranges remained constant due to no change in cylinder parameters. This resulted in a total of 10 trials per joint for this sensitivity analysis.

#### 2.3.5 SA5: Reducing mesh resolution

The original CT data were re‐segmented then meshed at the highest resolution feasible for the scan (i.e. faces ~voxel size), which generated a tibiotarsus mesh with 422,466 faces, a tarsometatarsus mesh with 220,417 faces and a phalanx III‐1 mesh with 35,753 faces. These were then subsequently downsampled to resolutions of 0.5 ×, 0.25 ×, 0.125 ×. An additional model containing less than 10,000 faces was also used, as recommended for the APSE method (Bishop et al., 2022), though we note that 10,000 faces could be achieved with a higher density mesh if parts of the bone away from the joint are cropped away. Bone meshes of all five resolutions were then assembled into the reference poses, with new articular surfaces selected for each resolution based on the morphology visible in that mesh. The number of faces for each mesh and joint at the different resolutions tested are detailed in Tables S1 and S2.

### 2.4 ROM analysis

6DOF ROM analysis was performed for each trial using the Blender ROM Finder add‐on (https://github.com/pfalkingham/BlenderROMFinder). All analyses were performed on a workstation equipped with an Intel Xeon processor (24 cores, 2.30Ghz) and 64GB RAM. The joint's motion space is evaluated in parallel, with each process stepping through its assigned rotations and translations sequentially and performing collision checks at each step (initially against a convex hull, and then in case of hulls colliding, the full mesh is checked). Non‐interpenetrating poses are determined as viable. Joint rotations are quantified using Euler/Tait‐Bryan angles, as recommended by the International Society of Biomechanics (Wu et al., 2002), with Z as the primary axis in an XYZ hierarchy. FE takes place about the ACSf Z‐axis, with 0° rotation representing a fully extended TMTP3 joint, positive values representing dorsiflexion and negative values representing plantarflexion. For the ankle, 180° FE represents a fully extended joint as rotations occur from the reference pose, rather than a zero pose as in the TMTP3 joint. ABAD takes place about the ACSm Y‐axis, where adduction is positive, and LAR takes place about the ACSm X‐axis where external rotation is positive. These axes remain static relative to the bones they are fitted to. This differs slightly from Gatesy et al. (2022), in which ABAD rotates around a floating Y‐axis derived from both ACSf and ACSm in a joint coordinate system (JCS). As we are solely using a hinge joint in the present study, ACSm Y remains perpendicular to ACSm X and Z; therefore, a floating axis was deemed unnecessary, so all rotations occur around the ACSs rather than a JCS. This process allows translations to be sampled at the ACSm, which is equivalent to the prism‐based hinge joint method proposed by Manafzadeh and Gatesy (2021) where translations occur from the JCS prior to rotations.

Each test was performed at 10° increments, with each rotational pose having a possible 125 translations (5 × 5 × 5). The tested rotation ranges were –90° to 90° for both X‐ and Y‐axes, and –180° to 180° for the Z‐axis. Although poses beyond the X and Y ranges may be viable, they were not tested since such poses would not be observed in vivo due to soft tissue constraints (Kambic et al., 2014). Translation ranges were determined by tarsometatarsus cylinder radius and height for the TMTP3 and tibiotarsus cylinder radius and height for the ankle to ensure they were relative to the size of the bone meshes used without the need for in situ joint spacing data. These ranges are intentionally large so that no rotational configurations used in life would be incorrectly excluded (see Data S1). Whilst other approaches have recorded only the first viable translational configuration (Manafzadeh et al., 2024), or the most optimal one (Demuth et al., 2025), we recorded all viable configurations to understand the full extent of the possible sensitivity. Smaller rotational increments and more possible translations would provide more detailed results, as is standard in 6DOF digital ROM analysis, however, the present ranges were deemed suitable for a sensitivity analysis as they will still reveal differences in ROM analysis outcomes and be comparable between trials. If results revealed a non‐continuous range of viable poses along an axis, poses beyond the largest continuous range were excluded from analyses. The results of each ROM analysis were plotted onto 3D ROM maps with an alpha shape encompassing all rotational poses, as per Manafzadeh and Padian (2018), using a ROM Alpha Shape Plotter script (https://github.com/ajannel/ROM_Plotter) in MATLAB R2025a (MathWorks Inc., 2025). Each ROM map has been cosine‐corrected for 2D presentation (Manafzadeh & Gatesy, 2020). The total number of viable poses, ROM map alpha shape true volume (not cosine‐corrected), and maximum rotations of each axis were all measured as outcomes for each trial. Individual ROM maps (3D and 2D) and details on number of viable poses and alpha shape volumes for each individual trial can be found in the supplementary information. For SA2, multiple linear regression and second‐order polynomial regression using quadratic terms were performed in R version 4.5.3 (R Core Team, 2025) to test the significance of the selection size of each articular surface on both the number of viable poses and the alpha shape volume.

## 3 RESULTS

ROM analysis outcomes varied across the sensitivity analyses for both the TMTP3 and ankle joints. Summary results showing the ranges for each outcome measure are presented in Table 1 for the TMTP3 joint and Table 2 for the ankle joint. Detailed results for each sensitivity analysis and joint are described in the following subsections.

**TABLE 1:** TMTP3 joint range of motion analysis results, including number of viable poses, alpha shape volume and rotation ranges, for sensitivity analyses 1–5.

TMTP3	Viable poses		Alpha shape volume (°^3^)		Joint angle ranges (°)					
	Min	Max	Min	Max	FE min	FE max	ABAD min	ABAD max	LAR min	LAR max
SA1	43,810	61,158	2,658,833	3,225,667	−130 to 110	−140 to 110	−50 to 50	−50 to 60	−90 to 90	−90 to 90
SA2	7568	653,868	1,309,000	8,106,500	−120 to 90	−150 to 160	−40 to 40	−90 to 90	−90 to 90	−90 to 90
SA3	46,210	46,827	2,790,833	2,860,333	−130 to 110	−140 to 110	−50 to 50	−50 to 50	−90 to 90	−90 to 90
SA4	45,665	47,946	2,784,833	2,886,500	−130 to 110	−140 to 110	−50 to 50	−50 to 50	−90 to 90	−90 to 90
SA5	39,865	47,765	2,667,667	2,893,000	−130 to 110	−140 to 120	−50 to 50	−40 to 60	−90 to 90	−90 to 90

Abbreviations: ABAD, abduction‐adduction; FE, flexion‐extension; LAR, long‐axis rotation; TMTP3, tarsometatarsophalangeal III.

**TABLE 2:** Ankle joint range of motion analysis results, including number of viable poses, alpha shape volume and rotation ranges, for sensitivity analyses 1–5.

Ankle	Viable poses		Alpha shape volume (°^3^)		Joint angle ranges (°)					
	Min	Max	Min	Max	FE min	FE max	ABAD min	ABAD max	LAR min	LAR max
SA1	5678	10,843	1,277,000	1,845,667	30 to 240	30 to 260	−60 to 60	−70 to 60	−90 to 90	−90 to 90
SA2	212	533,565	28,833	7,145,500	30 to 190	0 to 360	−70 to 60	−90 to 90	−10 to 50	−90 to 90
SA3	5540	7511	1,174,330	1,604,167	30 to 230	30 to 250	−70 to 60	−70 to 60	−90 to 90	−90 to 90
SA4	5680	7988	1,166,000	1,512,333	30 to 230	30 to 250	−60 to 60	−70 to 80	−90 to 90	−90 to 90
SA5	5219	7066	1,225,167	1,415,000	40 to 230	30 to 250	−50 to 50	−50 to 50	−90 to 90	−90 to 90

Abbreviations: ABAD, abduction‐adduction; FE, flexion‐extension; LAR, long‐axis rotation.

### 3.1 SA1: Changing size of articular surface selection

#### 3.1.1 TMTP3

For the TMTP3 joint, cylinder radius, and therefore X and Y translation ranges varied by 0.8% across trials. Cylinder height and Z translation ranges varied by 12.7%. The number of viable poses varied by 17,348 across trials, representing −5.5% and + 31.5% difference compared with the value for “normal” articular surface selection size for both bones Figure 2a. This suggests that increasing the articular surface selection size has an increasingly positive effect on the number of viable poses, although there were small exceptions. On the phalanx III‐1, a larger selection area resulted in a more proximally positioned plane due to the concave contours of the articular surface. As the centre of the plane determines the ACSm position, this caused a greater joint spacing; therefore, more poses were viable at lower X translations.

**FIGURE 2:**
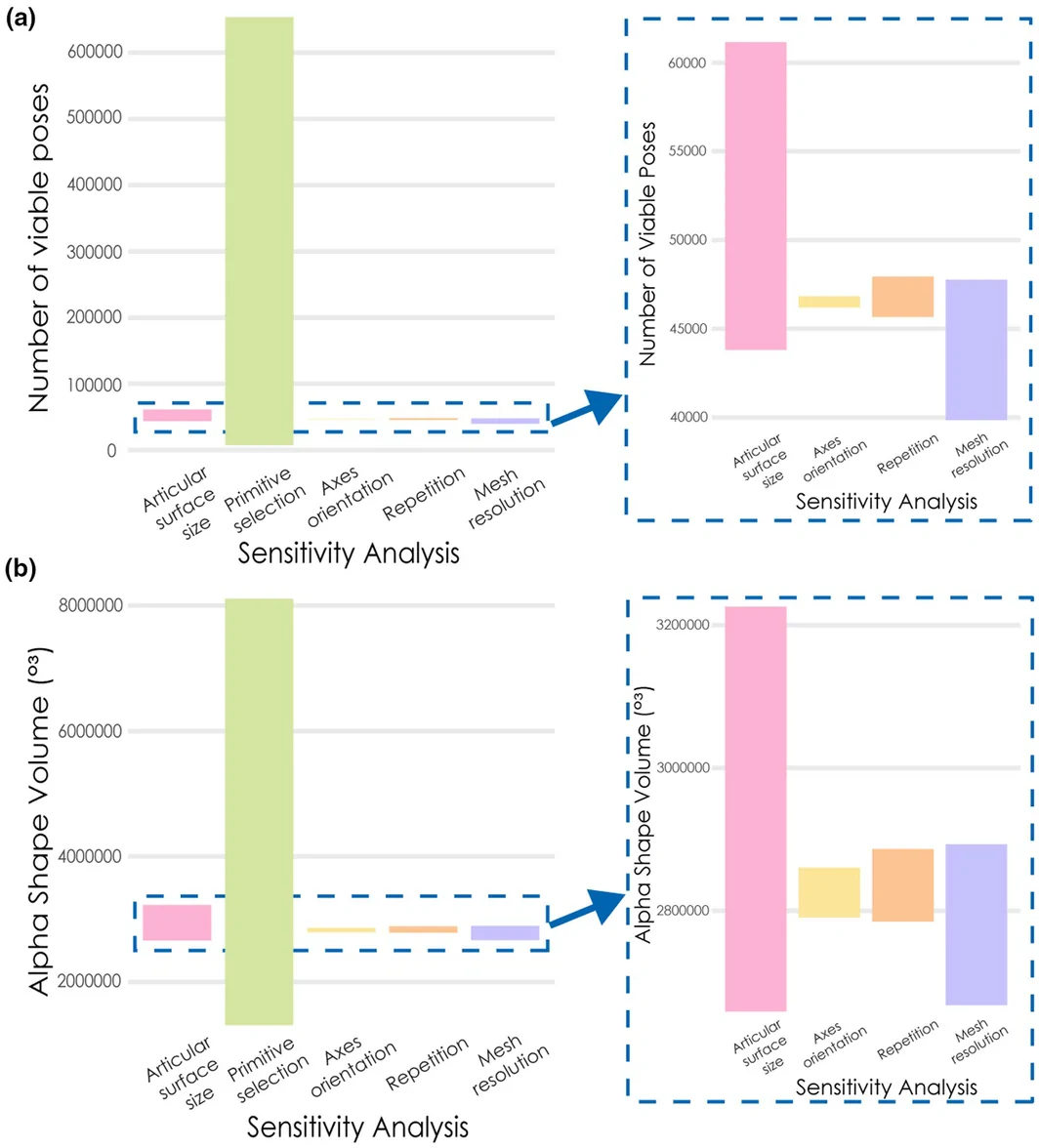
(a) Range of number of viable poses for each sensitivity analysis for the TMTP3 joint. (b) Alpha shape volume in°^3^ of the 3D ROM maps for each sensitivity analysis. Graphs on the right‐hand side exclude Primitive (SA2) for enhanced visualisation of differences between the other sensitivity analyses.

Alpha shape volume generally followed the same trend, consistently increasing as articular surface selection size increases, although to a lesser extent than the number of viable poses (Figure 2b). Volume varied by 566,833°^3^, representing −6.0% and + 14.0% of the “normal” values. Despite this, maximum viable rotations for each axis were generally consistent across trials (Figure 3a). An additional 10° of adduction was viable in trials where phalanx III‐1 selection was +1 or +2 and tarsometatarsus selection was +1 or +2, as well as when a + 1 phalanx III‐1 selection was combined with a normal tarsometatarsus selection. Trials with −2 or +2 tarsometatarsus selection size, combined with normal or smaller phalanx III‐1 selection, allowed for 10° more plantarflexion (see Table 1).

**FIGURE 3:**
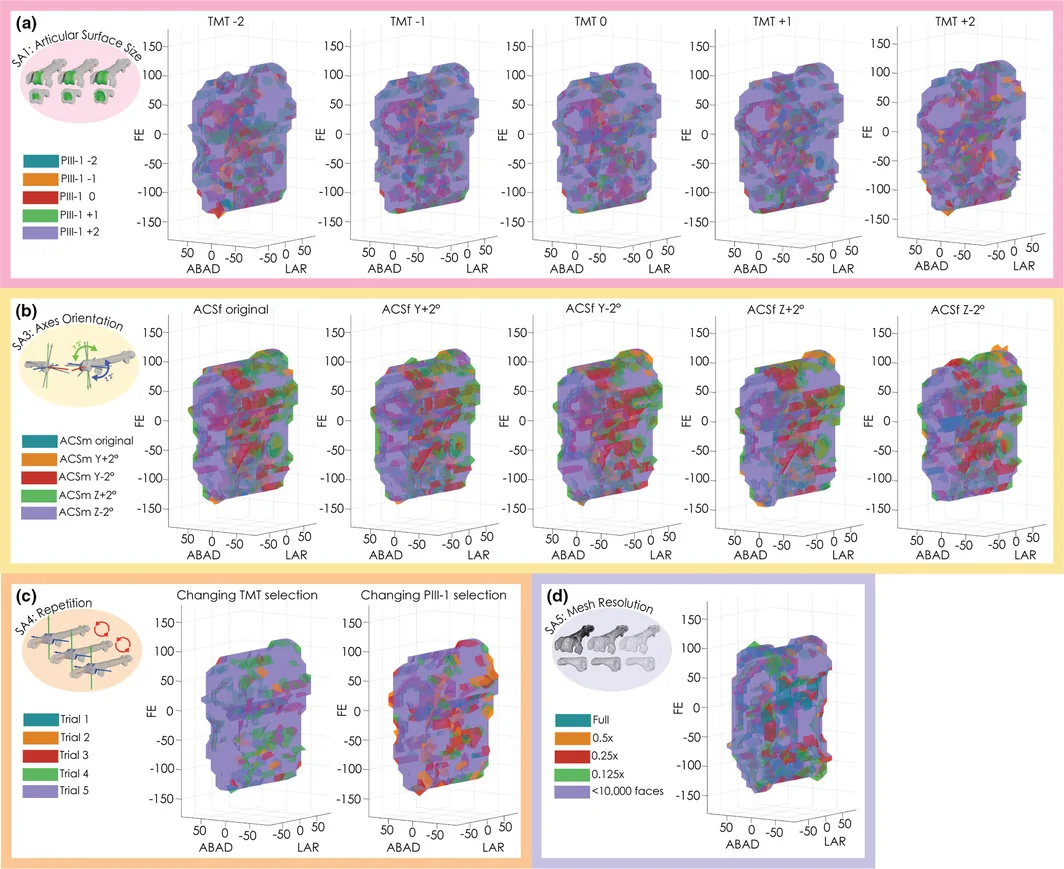
Cosine‐corrected 3D ROM maps of the TMTP3 joint show relatively minor changes across most sensitivity analyses. Axes show flexion/extension (FE), abduction/adduction (ABAD) and long‐axis rotation (LAR) angles in degrees at 10‐degree resolution for sensitivity analyses (a) SA1: Articular surface size, (b) SA3: Axes orientation, (c) SA4: Repetition and (d) SA5: Mesh resolution. Alpha shapes contain the full translation range. TMT, tarsometatarsus; PIII‐1, phalanx III‐1.

About 99% of the variation in results of all outcome measures can be explained by the articular surface selection size (*R* ^2^ = 0.99, *p* < 0.001). Both tarsometatarsus and phalanx III‐1 selection size had positive linear and quadratic effects on the number of viable poses (*p* < 0.001). Phalanx III‐1 selection size had a stronger influence on the number of viable poses than tarsometatarsus selection size. There was no significant interaction between tarsometatarsus selection size and phalanx III‐1 selection size (*p* = 0.33). For the alpha shape volume, tarsometatarsus selection size had a stronger linear effect (*p* < 0.001) than the phalanx III‐1 selection size (*p* < 0.001), and the phalanx III‐1 then had a stronger curvilinear effect (*p* < 0.001) than the tarsometatarsus (*p* < 0.01). This suggests that the tarsometatarsus selection size has a greater influence on alpha shape volume at lower levels, whereas the influence of the phalanx selection size continues to increase as the sizes move toward the extremes. No interaction was observed between the tarsometatarsus and phalanx III‐1 selection sizes (*p* = 0.48).

#### 3.1.2 Ankle

Unlike the TMTP3, the ankle showed an inconsistent response to articular surface selection size change, with a larger overall variation in ROM analysis outputs (Figure 4). Cylinder radius, and therefore X and Y translation ranges varied by 2.2%. Cylinder height and Z translation ranges varied by 8.0%. The number of viable poses varied by 5145, representing −21.6% and +49.7% difference from the value for “normal” articular surface selection size for both bones (Figure 4a). Similarly to the TMTP3, as tarsometatarsus articular surface selection size increased, the number of viable poses increased. However, changing the tibiotarsus selection size did not have the same effect; the highest number of poses were generally produced when the selection size was −2, and the lowest produced when the selection size was +2, although this was inconsistent and the trend varied between sizes. The overall highest number of viable poses was produced with the combination of a +1 tibiotarsus articular surface and a + 2 tarsometatarsus articular surface selection size, and the lowest was produced by the combination of +2 tibiotarsus and –2 tarsometatarsus articular surfaces.

**FIGURE 4:**
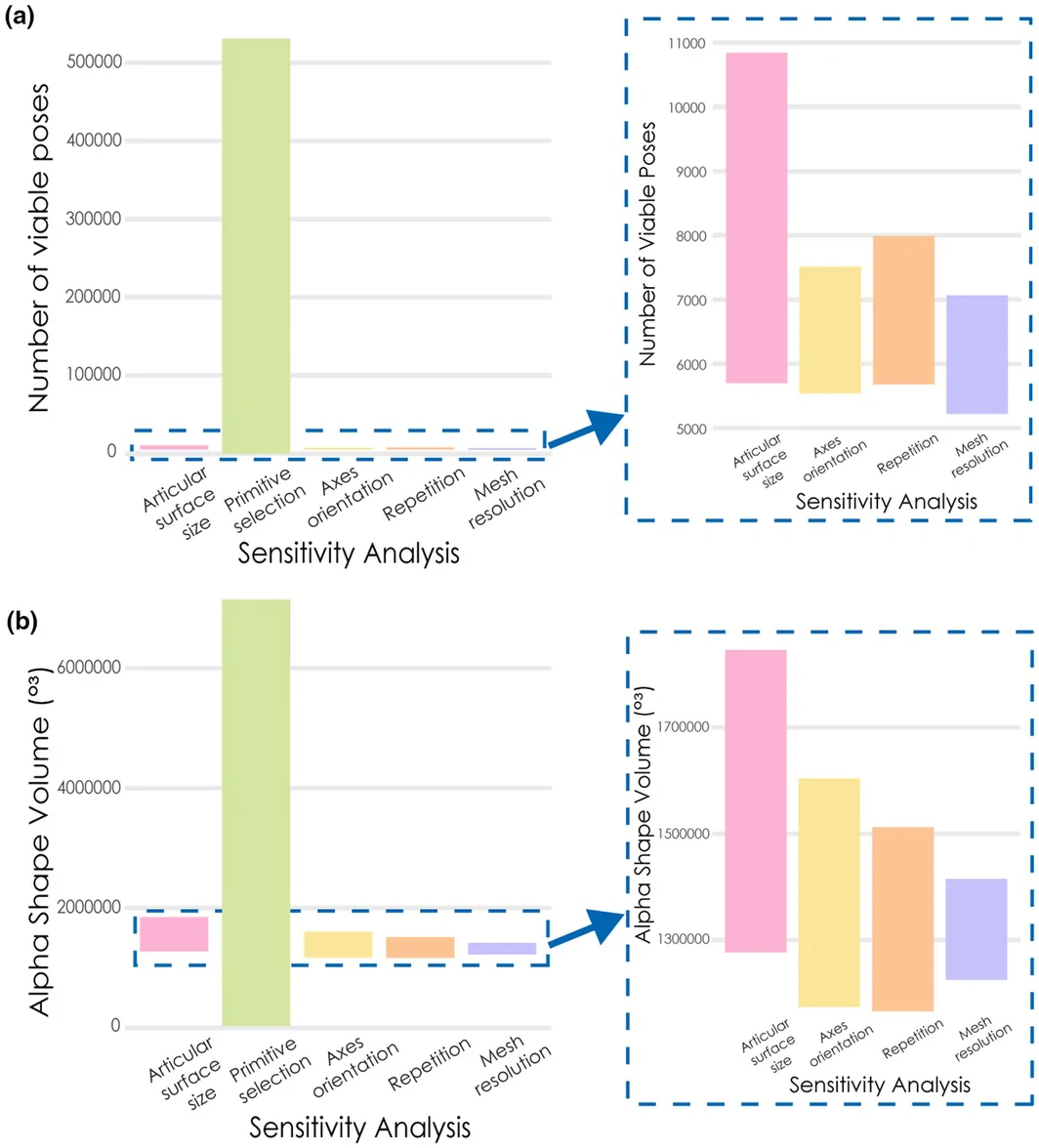
(a) Range of number of viable poses for each sensitivity analysis for the ankle joint. (b) Alpha shape volume in°^3^ of the 3D ROM maps for each sensitivity analysis. Graphs on the right‐hand side exclude Primitive (SA2) for enhanced visualisation of differences between the other sensitivity analyses.

Alpha shape volume varied by 568,667°^3^, representing −10.3% and +29.7% difference from the “normal” values (Figure 4b). As tarsometatarsus articular surface selection size increased, the alpha shape volume generally increased, although this was inconsistent. Changes in tibiotarsus articular surface selection size produced irregular changes to alpha shape volume with no consistent directional pattern. Extension increased by 10° when +1 or +2 tarsometatarsus selection sizes were combined with −1 or larger tibiotarsus selection sizes, and when normal tarsometatarsus selection was combined with +1 or +2 tibiotarsus selection, except for the combination of +2 tibiotarsus and +2 tarsometatarsus which had one pose at 20° more extension (Figure 5a). Maximum ABAD rotations remained relatively consistent, with three trials having 10° less abduction and one of these trials also having 10° less adduction. Maximum LAR ranges remained consistent across trials (see Table 2).

**FIGURE 5:**
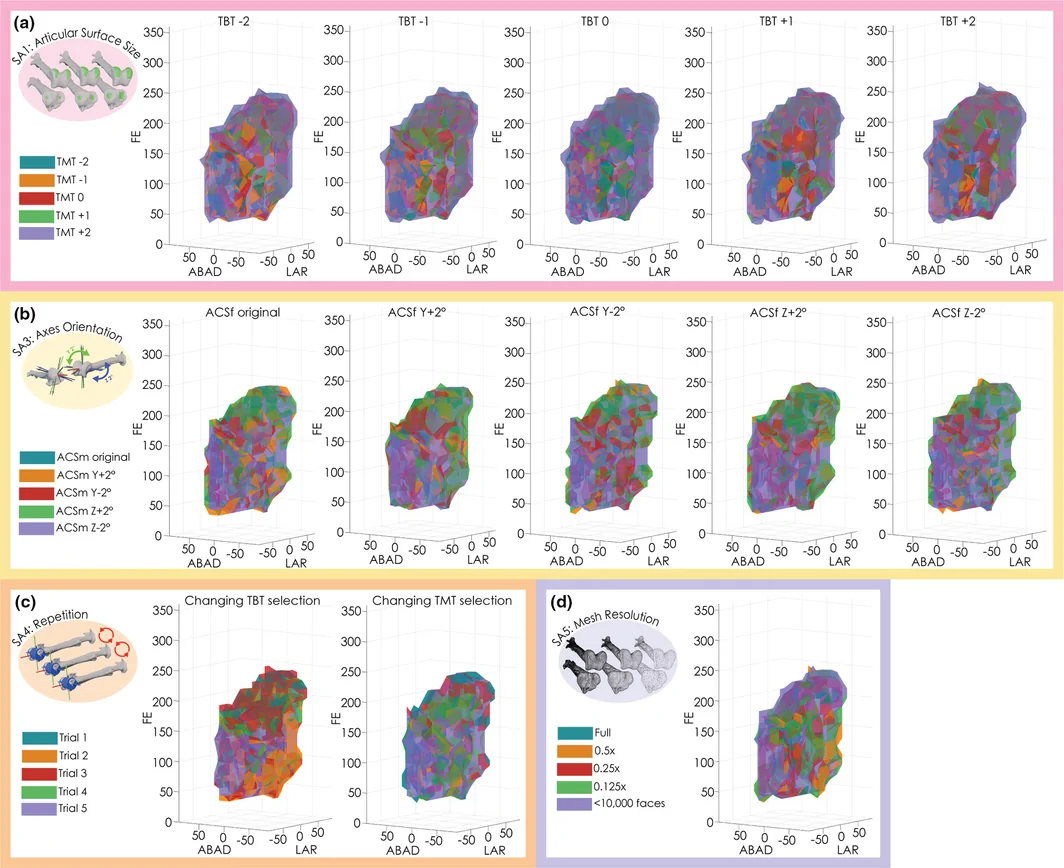
Cosine‐corrected 3D ROM maps of the ankle joint show slightly more variation between trials for most sensitivity analyses than the TMTP3 joint. Axes show flexion/extension (FE), abduction/adduction (ABAD), and long‐axis rotation (LAR) angles measured in degrees at 10‐degree resolution for sensitivity analyses (a) SA1: Articular surface size, (b) SA3: Axes orientation, (c) SA4: Repetition, and (d) SA5: Mesh resolution. Alpha shapes contain the full translation range. TBT, tibiotarsus; TMT, tarsometatarsus.

For the number of viable poses, a second‐order polynomial model explained 96% of the variation (*R* ^2^ = 0.96, *p* < 0.001). Tarsometatarsus selection size showed a significant positive linear and quadratic effect on the number of viable poses (*p* < 0.001), indicating a curvilinear increase in viable poses as the selection size increases. Tibiotarsus selection size showed a significant linear effect (*p* = 0.002) but no significant quadratic effect (*p* = 0.28) and the interaction between tibiotarsus and tarsometatarsus selection sizes was not significant (*p* = 0.72). For alpha shape volume, the model explained 84% of the variation (*R* ^2^ = 0.84, *p* < 0.001). Similarly to the number of viable poses, tarsometatarsus selection size had a significant positive linear effect on alpha shape volume (*p* < 0.01), whereas tibiotarsus selection size was not significant (*p* = 0.57). There was no interaction between tibiotarsus and tarsometatarsus selection sizes (*p* = 0.13). Quadratic terms were not supported, indicating no evidence of curvilinear effects on alpha shape volume.

### 3.2 SA2: Changing primitive

#### 3.2.1 TMTP3

Changing the primitive caused the largest variation in ROM analysis outcomes out of all the tested sensitivity analyses. For the TMTP3 joint, changing the primitive fitted to the proximal end of phalanx III‐1 had a greater effect than changing the primitive fitted to the distal end of the tarsometatarsus, with phalanx III‐1 cylinders causing the highest results of all outcome measures regardless of the tarsometatarsus primitive. The number of viable poses ranged from 7568 (sphere–sphere combination) to 653,868 (tarsometatarsus plane‐phalanx III‐1 cylinder combination), with a mean of 234,050.89 and a median of 51,119 (Figure 2a). Alpha shape volume also varied greatly due to the large differences in viable pose space and maximum rotations. Volume ranged from 1,309,000°^3^ (sphere–sphere combination) to 8,106,500°^3^ (tarsometatarsus cylinder‐phalanx III‐1 cylinder combination), with a mean of 4,219,093°^3^ and a median of 2,829,000°^3^ (Figure 2b).

Fitting a cylinder to the proximal end of phalanx III‐1 caused an increase in the maximum rotations, allowing full −90° to 90° ABAD and LAR and increasing FE to −150° to 160° with a tarsometatarsus cylinder and sphere, and to −150° to 140° with a tarsometatarsus plane (Figure 6). This appeared to be caused by the placement of the cylinder, as one side of the cylinder was automatically positioned at the articular surface. Therefore, the centre of rotation was positioned proximally to the bone, causing extreme joint space at the reference pose and all tested joint configuration due to the translation ranges remaining constant. This allowed for viable poses at all X translations, increasing the results of all outcome measures. Using a sphere for the proximal articular surface of the phalanx III‐1 had the opposite effect; the centroid of the sphere was positioned distally to the surface, reducing the joint space. This meant that only poses at the two largest possible X translations were valid, reducing the results of all outcome measures.

**FIGURE 6:**
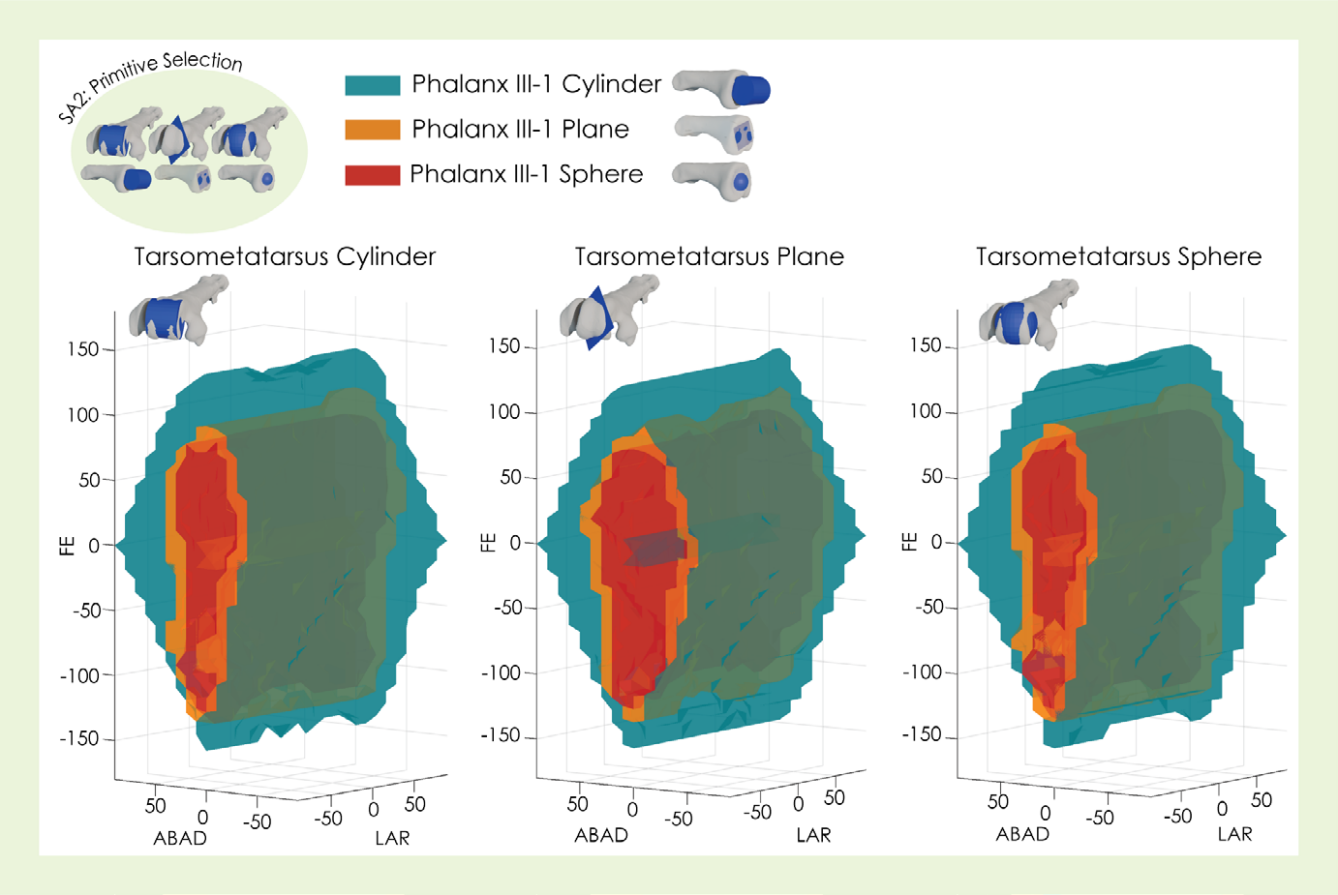
Cosine‐corrected 3D ROM maps of the TMTP3 joint showing flexion/extension (FE), abduction/adduction (ABAD), and long‐axis rotation (LAR) angles measured in degrees at 10 resolution for SA2: Changing primitive. Alpha shapes contain the full translation range.

There was little variation in poses between fitting a cylinder or sphere to the distal end of the tarsometatarsus; however, a plane resulted in a higher number of viable poses and greater alpha shape volume. The centroid of the plane was positioned more distally than other primitives, closer to the articular surface. This generated more joint space and allowed for viable poses at all X translations; hence, the high number of viable poses. This had less of an effect than when fitting a cylinder to the phalanx III‐1, as it only increased ABAD by up to 20°, and reduced FE by 10–20° when combined with a phalanx III‐1 cylinder or sphere. The highest number of viable poses resulted from the combination of a plane fitted to the tarsometatarsus and a cylinder fitted to the phalanx III‐1. Interestingly, this combination did not have the greatest maximum angles, due to 20° less dorsiflexion than when fitting a cylinder or sphere to the tarsometatarsus in combination with a phalanx III‐1 cylinder. Therefore, the greatest alpha shape volume resulted from cylinders fitted to both the tarsometatarsus and the phalanx III‐1 (see Table 1).

#### 3.2.2 Ankle

Similar to the TMTP3 joint, ankle ROM outcomes were more affected by changes to the distal bone primitive (on the proximal end of the tarsometatarsus) than changes to the proximal bone primitive (on the distal end of the tibiotarsus). The number of viable poses ranged from 212 (tibiotarsus cylinder‐tarsometatarsus sphere combination) to 533,565 (plane‐cylinder combination), with a mean of 143,538 and a median of 36,672 (Figure 4a). Alpha shape volume also varied greatly, ranging from 28,833°^3^ (tibiotarsus cylinder‐tarsometatarsus sphere combination) to 7,145,500°^3^ (cylinder–cylinder combination), with a mean of 3,591,630°^3^ and a median of 1,423,333°^3^ (Figure 4b).

As observed for the TMTP3 joint, fitting a cylinder to the proximal end of the tarsometatarsus caused a large increase in the viable rotational pose space and number of viable poses, enabling viable poses at the full rotational search range (Figure 7). Once again this is due to the centroid of the cylinder being positioned proximal to the articular surface, causing extra joint space. The sphere fitted to the proximal end of the tarsometatarsus was visually very large, with the centre positioned distal to the articular surface, so there was less joint space and therefore very few viable poses compared with trials with other primitives. When the tarsometatarsus sphere was combined with a tibiotarsus plane or sphere, ABAD was restricted to high abduction values. There was little variation in the measured outcomes between fitting a cylinder or sphere to the distal end of the tibiotarsus; however, the shape of the ROM maps visually differed, showing variation in the occupied rotational pose space. The plane fitted to the tibiotarsus was positioned more anteriorly than the other primitives. This enabled full −90° to 90° ABAD and a high number of viable poses regardless of the tarsometatarsus primitive type. However, as it also limited FE to <250°, alpha shape volume was lower for the tibiotarsus plane‐tarsometatarsus cylinder combination compared with the combination of other tibiotarsus primitives with the tarsometatarsus cylinder (see Table 2).

**FIGURE 7:**
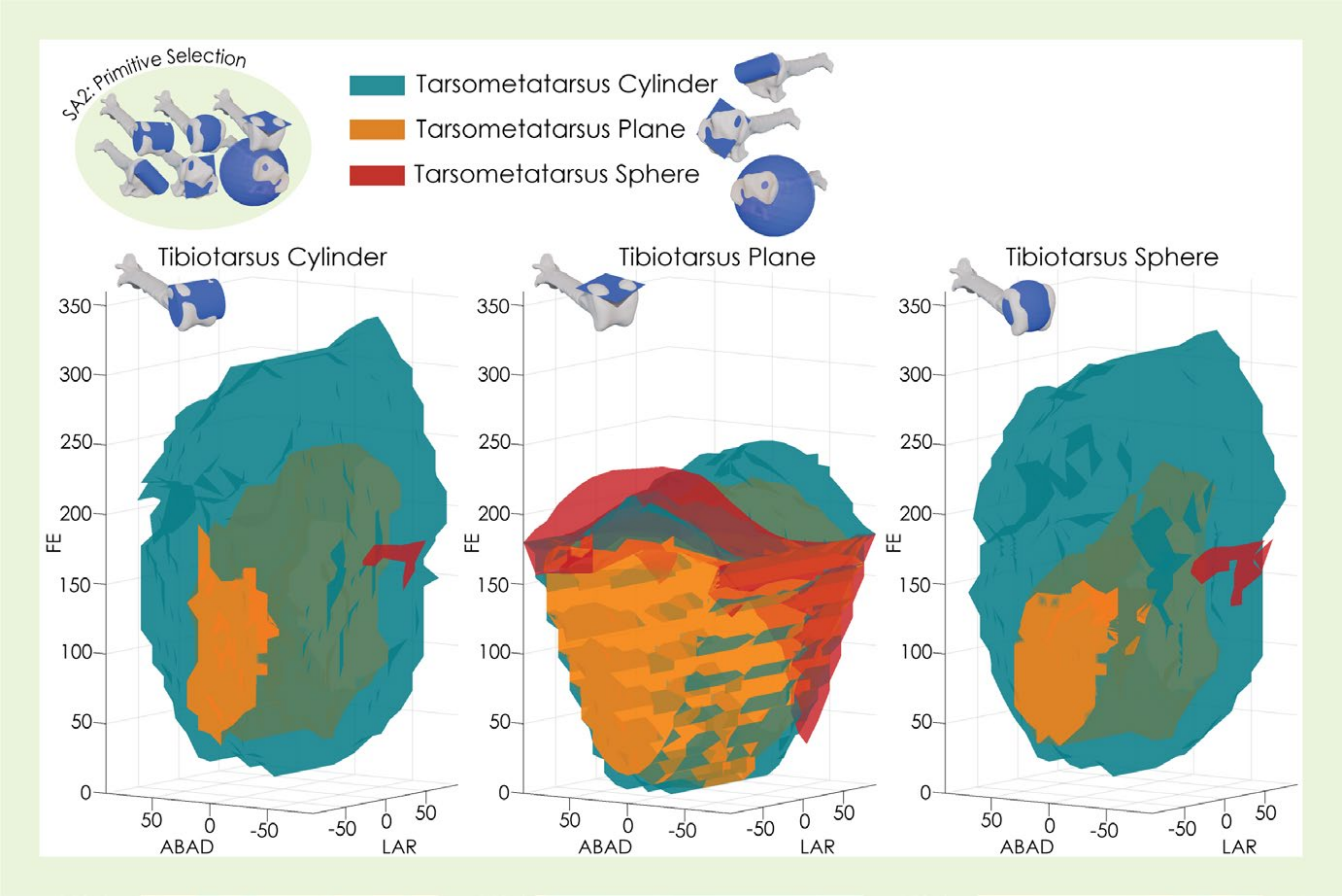
Cosine‐corrected 3D ROM maps of the ankle joint showing flexion/extension (FE), abduction/adduction (ABAD) and long‐axis rotation (LAR) angles measured in degrees at 10‐degree resolution for SA2: changing primitive. Alpha shapes contain the full translation range.

### 3.3 SA3: Changing axes orientation

#### 3.3.1 TMTP3

For the TMTP3 joint, the total variation in number of viable poses across all orientations was 617, which represents a difference of −0.7% to +0.7% compared with the value for the original orientations, indicating that axis orientation only had a small effect overall (Figure 2a). ACSm orientation appears to have a greater influence on number of viable poses than ACSf, particularly the Y‐axis. Rotating the ACSm Y‐axis −2° resulted in the highest number of viable poses and rotating it +2° produced the lowest, regardless of the ACSf orientations. This axis orientation is dependent on the location of the cylinder fitted to the distal phalanx III‐1. A more laterally placed cylinder would have the effects of a negatively rotated Y‐axis, whereas a medially positioned cylinder would have the effects of a positively rotated Y‐axis.

There was a greater effect on alpha shape volume; however, this was still minimal with a total variation of 69,500°^3^, representing −1.3% to +1.1% compared with the original orientations (Figure 2b). Volume was generally more affected by changing ACSm Z rather than ACSm Y, with the greatest volume value resulting from rotating ACSm Z + 2° and the lowest caused by −2° rotation. This kind of rotation would be caused by the cylinder on the distal phalanx III‐1 being positioned more cranially or caudally. ABAD and LAR remained consistent throughout trials; however, FE varied by 20° (Figure 3b). These results suggest that axes orientation only has a minor effect on poses for the TMTP3 joint (see Table 1).

#### 3.3.2 Ankle

Changing axes orientation in the ankle joint had a greater effect on ROM analysis results; the number of viable poses varied by 1971 across all orientations and there was a difference of −23.5% and + 3.7% compared with the value for the original orientations (Figure 4a). Unlike the TMTP3 joint, rotating the ACSf had a greater effect on the determined viable poses than ACSm. Rotating the ACSf Y‐axis +2° consistently produced the lowest number of viable poses, whereas rotating this axis −2° consistently increased the number of viable poses slightly. Alpha shape volume was slightly more affected than the number of viable poses, varying by 429,837°^3^ and differing from the original orientations by −17.5% and +12.7% (Figure 4b). Changing the ACSm Y‐ or Z‐axes–orientation resulted in a lower alpha shape volume when combined with normal, Y + 2° or Z – 2° ACSf orientations, whereas it resulted in higher volume when combined with ACSf Y – 2° or Z + 2°. Maximum FE rotations across all orientations varied by 30° and ABAD varied by 10° (Figure 5b). Maximum LAR rotations remained constant (see Table 2).

### 3.4 SA4: Repetition of articular surface selection

#### 3.4.1 TMTP3

When changing the tarsometatarsus selection, cylinder radius, and therefore X and Y translation ranges, varied by 1.0%, whilst cylinder height and Z translation ranges varied by 4.0%. Across these trials, mean number of viable poses was 46,581 with a standard deviation of ±287.59 (Figure 2a) and the mean alpha shape volume was 2,835,100°^3^ ± 14,079°^3^ (Figure 2b). Maximum viable LAR and ABAD rotations remained constant across all trial. FE remained generally consistent, however two of the five trials had a single pose at 10° more plantarflexion (Figure 3c). When changing the phalanx III‐1 selection, translations ranges remained constant as the cylinder was not affected. The mean number of viable poses was 46785.40 with a standard deviation of ±743.86 (Figure 2a) and the mean alpha shape volume was 2,842,933°^3^ ± 36,532°^3^ (Figure 2b). Maximum rotations were the same as when changing the tarsometatarsus selection, again with two trials having one pose at 10° more plantarflexion than the other trials (see Table 1).

#### 3.4.2 Ankle

Repetition of the articular surface selections in the ankle reference pose assembly process resulted in greater variation of ROM analysis results than the TMTP3. When changing the tibiotarsus articular surface, cylinder radius, and therefore X and Y translation ranges varied by 5.2%, whilst cylinder height and Z translation varied by 4.8%. The mean number of viable poses was 6869.40 ± 720.19 (Figure 4a) and the mean alpha shape volume was 1,348,933°^3^ ± 124,868°^3^ (Figure 4b). Unlike the TMTP3, changing the articular surface of the distal bone resulted in less variation than changing that of the proximal bone. When changing the tarsometatarsus articular surface selection, the mean number of viable poses was 6397.80 ± 585.12 (Figure 4a) and the mean alpha shape volume was 1,420,667°^3^ ± 54,684°^3^ (Figure 4b). Maximum rotations of each axis varied more for the ankle than for the TMTP3, with a total of 30° variation of ABAD and 20° FE (Figure 5c). LAR remained consistent (see Table 2).

### 3.5 SA5: Changing mesh resolution

#### 3.5.1 TMTP3

There was a 19.8% total increase in the number of viable poses between full resolution and <10,000 faces, although there was a slight decrease of 407 (1.0%) poses between resolutions of 0.5 × and 0.25 × (Figure 2a). Alpha shape volume increased by 8.5% between maximum and minimum resolution (Figure 2b). This mostly increased continuously as mesh resolution decreased; however, there was a decrease of 3.2% volume between 0.25 × and 0.125 × resolution. Plantarflexion increased by 10° at all lower resolutions, and 10° more dorsiflexion was possible at 0.125 × resolution (Figure 3d). Adduction increased by 10° at both 0.5 × resolution and <10,000 faces (see Table 1).

#### 3.5.2 SA5: Ankle

The effect of mesh resolution on the ankle differed from the TMTP3 joint. Whilst the lowest resolution (< 10,000 faces) did result in a higher number of viable poses and slightly larger alpha shape volume than the highest resolution (increases of 11.3% and 0.03% respectively), there was no consistent trend between resolutions (Figure 4). The lowest number of viable poses and lowest alpha shape volume was produced with 0.25 × resolution. The highest number of viable poses was produced with 0.5 × resolution, although the highest alpha shape volume was produced with 0.125 × resolution. Maximum FE varied by a total of 30° between resolutions; however, there was no pattern to this variation between resolutions (Figure 5d). Maximum ABAD and LAR rotations remained constant (see Table 2).

#### 3.5.3 SA5: Processing time

Decreasing the resolution decreased the time taken to run the ROM analysis for both joints. When testing at the full rotation range (1,669,625 poses tested), the highest resolution for the TMTP3 joint (256,170 faces) took 20:46 min whereas the smallest resolution (9604 faces) was completed in 4:37 min. For the ankle, the highest resolution (642,883 faces) took 22:21 min and the lowest resolution (9664 faces) took 5:20 min. These times are obviously dependent on computer hardware (see Methods for specifications), but the magnitude of difference is indicative of the time savings a lower mesh provides. Comparing this to the limited sensitivity of outcome measures between resolutions reveals a trade‐off between analysis time and relatively small changes in outputted viable poses.

## 4 DISCUSSION

ROM analysis has become more widely used in palaeontology, where in vivo or ex vivo validation is not possible. There has been a trend in palaeontological research for some time to move towards more objective methods, which is reflected in the development of new ROM analysis methods. The automated 6DOF method provides an objective way of sampling all rotational and translational configurations to determine viable poses. However, the sensitivity analysis presented here reveals that this objectivity is compromised by the subjective processes involved in the reference pose assembly, including primitive choice and articular surface selection, as objective results cannot be produced from subjective input data (Falkingham, 2016). This is not necessarily something that can be 'solved’, merely the result of applying objective computer processes to messy and variable biological data.

Changing the primitive used to determine joint centre has the greatest overall effect on the viable poses determined by ROM analysis for both tested joints, including causing large differences in occupied rotational pose space and maximum FE and ABAD angles. This aligns with previous preliminary sensitivity analyses, in which osteological joint mobility was found to significantly vary depending on the primitive shape used to determine a joint centre (Demuth et al., 2020). Some of this variation is derived from the decision to test consistent translation ranges between trials despite variance in joint spacing at the reference/zero pose caused by the primitive type. This means that for analyses with larger initial joint spacing, such as when fitting a cylinder to the distal articular surface of the proximal bone, the distal bone visually appears to have translated further away from the proximal bone despite having moved the same amount from zero. This could result in poses becoming viable at the same rotational and translational configurations as non‐viable poses for models with less initial joint space. The reverse will also be true for reference/zero poses with smaller initial joint spacing, such as when fitting a sphere to the proximal articular surface of the tarsometatarsus in the ankle joint, resulting in very few viable poses. The significant variation caused by changing the primitives suggests that all future ROM analyses of tetrapod hindlimbs should follow the standard reference pose assembly proposed by Gatesy et al. (2022), in which cylinders are fitted to the distal articular surfaces and planes to the proximal articular surfaces for hinge joints, which would enable comparable results across studies. Exceptions to this can occur in cases of extreme morphological variance where alternate primitives may be better suited; however, explicit explanation and justification should be made in these cases. All primitives should be visually checked to determine if they are an appropriate fit, rather than blindly relying on automated fitting.

ROM analysis is highly sensitive to small changes in the articular surface, making it unlikely that two independent researchers would obtain identical results even when using the same primitives. In our cases, pose viability is more sensitive to changes in the distal bone articular surface selection area than the proximal bone selection. This is likely due to the concave morphology of the proximal articular surfaces of the phalanx III‐1 and tarsometatarsus, as slight changes in vertex selection can cause the fitted plane to be positioned more proximally or distally, altering joint spacing and centre of rotation. Articular surface selection determines the size and orientation of the primitives, which in this method also influences translation ranges. For the TMTP3 joint, increasing the size of either articular surface causes an increase in the number of viable poses and alpha shape volume, with the rate of increase becoming progressively greater at larger selection sizes. However, for the ankle, whilst changing the size of the distal bone articular surface followed this trend, increasing the size of the articular surface of the proximal bone beyond the normal selections often decreased the number of viable poses, occasionally more so than when reducing the selection size. This highlights the importance of aiming to select the articular surface based on the area that would be covered by articular cartilage, using selections suggested by Gatesy et al. (2022) as a guide. However, as shown by SA4, it is difficult even for a trained eye to be consistent with the selection, therefore we recommend that authors perform their own sensitivity analysis for this step. This also stresses the value of sharing all data including articular surface selections, reference poses and zero poses alongside published results as ROM analysis outcomes are model‐specific.

Articular surface selection determines the size and orientation of the primitives, which in this method also influences translation ranges. The dependency on the articular surface selection for translation ranges could be partially mitigated by using a fixed translation range based on in situ articular cartilage thickness measurements rather than primitive parameters, although these may be difficult to obtain, particularly if performing ROM analyses on extinct taxa. Whilst measurements from the taxa forming the extant phylogenetic bracket (Holliday et al., 2010; Witmer, 1995) could be utilised for extinct taxa, this approach may lead to inaccurate assumptions of the true joint space, particularly in larger species (Holliday et al., 2010; Scheidt et al., 2026). Other studies have found that articular cartilage morphology differs from the underlying bone, so simply applying a set cartilaginous thickness may be insufficient when attempting to account for the loss of this soft tissue (Bonnan et al., 2010; Voegele et al., 2022). It has been suggested that the morphological difference is greater for bones not utilised during locomotion and therefore may be less of a concern when simulating the mobility of hindlimb joints (Bonnan et al., 2010). Whilst changing axes orientation (SA3) had little effect for the TMTP3, it caused the third most variation in alpha shape volume out of all the sensitivity analyses for the ankle joint, mostly reducing the volume compared with the normal axes orientations. Small changes in axes orientation mean that the bones are positioned slightly differently in each joint configuration. For the ankle this altered the determined viable poses, as the curvature of each articular surface meant that the intercondylar eminence of the tarsometatarsus was not aligned into the intercondylar fossa of the tibiotarsus as normal, therefore colliding at configurations that were previously viable when using the original orientations. Finer rotation resolution sampling around these missed poses after the initial run could help mitigate this sensitivity, however, this would also drastically increase the number of tested poses, hence increasing computing time and ultimately producing very large datasets.

Mesh resolution also appears to affect ROM analysis outcomes, with lower resolution bone meshes generally resulting in a higher number of viable poses; however, this was more consistent for the TMTP3 joint than the ankle. Resolution of bone meshes can vary greatly depending on the scan parameters used (Balolia & Massey, 2021; Oakes et al., 2020). Morphological analysis of CT scanned primate skulls has shown that accurate anatomical measurements are only possible at higher resolutions, due to the loss of bone morphology at lower resolutions (Balolia & Massey, 2021). No study to date has tested how mesh resolution impacts digital ROM analysis results, despite APSE having a recommended maximum mesh size of less than 10,000 faces (Bishop et al., 2022). The results presented here for the TMTP3 joint suggest that lower resolution meshes generally enable greater rotational pose space to be occupied following ROM analysis, likely because loss of surface morphology reduces the bony prominences which would cause collisions. However, for the ankle, there was no consistent trend, with the highest number of poses being produced from the second highest resolution. As all new articular surfaces had to be selected, with unique primitives fitted for each model, the sensitivity apparent in these results is not solely due to resolution, as shown in the variation of SA4 results when using the same resolution for each trial. Whilst higher resolution bone meshes may more accurately represent the true morphology of the bones, there is a substantial trade‐off in how long the analyses take. Considering the limited sensitivity of the outcome measures to changes in mesh resolutions and the inconsistent trends observed, the relatively small differences in outputted viable poses are not proportional to the additional analysis time. This may mean that very high resolution meshes are not necessary for ROM analysis, depending on the research aims and computing power available. However, as ROM analysis is sensitive to articular surface selection size, a lower resolution mesh may increase the chances of an over‐ or under‐selected articular surface, affecting viable poses. Larger mesh faces may mean that small increases in the number of vertices selected will increase the articular surface size more than in higher resolution meshes. It could also cause faces on the articular surfaces to have steeper angles, which would mean that selections that are visually equal sizes could cause the primitives to be fitted at a different position along the long axis of the bones, affecting joint spacing. Therefore, higher resolution could enable greater precision when selecting the articular surface and fitting primitives, possibly reducing inter‐ and intra‐user variation.

For the TMTP3 joint, when the same reference pose assembly process is followed using the standard primitives (Gatesy et al., 2022), maximum rotational angles only vary slightly. Aside from changing the primitive, the other sensitivity analyses performed here had little effect on the maximum rotations of each axis, remaining generally consistent with maximum variations of 20° FE and 10° ABAD across all other trials when measured at 10° increments. The variation in results between researchers may be less substantial if maximum angles are the primary interest, which is common when trying to rule out anatomically impossible poses and behaviours in palaeobiology. Although for the ankle joint, maximum rotational angles were less consistent across trials, with variations of up to 30° FE and 30° ABAD (excluding SA2). Even when only reselecting one articular surface each time (SA4), extension varied by 20°, suggesting that the degree of sensitivity depends on the specific joint measured. Alpha shape volume also varies across trials for both joints, as the occupied rotational pose space varies regardless of changes in maximum rotational angles. This again highlights that this method is model‐specific, so all reference and zero poses, including articular surfaces, primitives, ACSf and ACSm axes, should be shared alongside results to ensure repeatability. Although both joints tested in this study are hinge joints, the sensitivity differed between them, with the ankle joint having greater variation in ROM analysis outcomes than the TMTP3 joint. One explanation for this could be the increased curvature of the proximal tarsometatarsus morphology compared with that of the phalanx III‐1. Greater curvature amplifies the impact of small changes in articular surface selection on primitive orientation, and consequently centre of rotation determination, hence the larger variation in results compared with the TMTP3 when repeating the articular surface selection (SA4). Given that hinge joints possess relatively simple morphology, other joint types with more complex morphology, such as ball‐and‐socket joints, would likely introduce even greater subjectivity during model construction. Therefore, the assumptions addressed here may be even more critical for proximal limb joints. As articular morphology varies significantly between clades and species, establishing a single set of standards to follow across all joint model constructions is impossible. Thus, whilst we recommend that future ROM analyses of tetrapod hindlimb joints follow the standards proposed by Gatesy et al., 2022, we strongly advise that authors also perform their own sensitivity analyses, even if only a subset of that presented here, regardless of joint type to account for these inherent variations.

6DOF ROM analysis results should be interpreted with caution, due to limitations in the method beyond the sensitivity in reference pose assembly presented here. Centroids of primitives fitted to the articular surfaces were found to inaccurately predict joint centre position when using an avian shoulder model, suggesting the joint axes derived from these may not accurately represent the true joint axes (Demuth et al., 2025). Although translational motions will help account for this by enabling a dynamic joint centre, the joint mobility determined from automated collision‐based 6DOF ROM analysis is unlikely to be the exact true joint mobility of the individual. It can, however, provide an insight into the possible joint mobility which can be compared across species. In vivo, soft tissues constrain maximum joint mobility to provide stability (Hutson & Hutson, 2012; Manafzadeh & Padian, 2018), therefore, osteological ROM analysis is likely to overestimate the rotational pose space occupied in vivo. This is particularly evident for LAR in the analyses presented here, which was viable at the full −90° to 90° range in most trials. In contrast, in vivo experiments have measured a maximum of approximately 20° ankle LAR (Kambic et al., 2014). Post hoc analysis methods have been proposed to constrain ROM analysis results to poses that would be seen in vivo, including raycasting in which viable poses are given an articulation score (Manafzadeh et al., 2024), thresholding poses based on overlap (Bishop et al., 2022), and ligament simulation (Demuth et al., 2025). It is possible that applying one of these post hoc methods to the ROM analysis results presented here would minimise the effects of variation in reference pose assembly, reducing the overall sensitivity.

## 5 CONCLUSION

The results presented here suggest that 6DOF ROM analysis is sensitive to inescapably subjective processes involved in the reference pose assembly, even if following strict protocols. Whilst this may be unlikely to directly affect the overall conclusions of any given individual study, it potentially makes comparison between studies and taxa problematic.

We found that changing the primitive type used to determine the joint centre of rotation produced the greatest amount of variation in outcomes, including large differences in maximum viable rotational angles. However, incorrect or subjective selection of primitive type is likely the least variable parameter between studies and researchers given the existing protocols (Gatesy et al., 2022). Only in particularly complex morphologies and niche edge cases is this likely to cause issues, and even then researchers will undoubtedly describe the primitive used in their methods.

Measured joint mobility is also sensitive to relatively small changes in articular surface selection size, and results vary even between repetitions of articular surface selection on the same bone, with the same researcher. This might be one of the greatest sources of variation between workers and studies and is also difficult to communicate via only figures and text within publications.

Sensitivity varied between the two joints studied here, even though both were hinge joints, so it is apparent that the degree of sensitivity of the subjective variables is model and joint specific. Our ankle joint appeared to be more sensitive than the TMTP3 joint, potentially due to increased curvature of the articular surfaces. More complex joints may naturally invite more subjectivity; therefore, we recommend that researchers perform their own sensitivity analyses for the specific joint tested.

Given the difficulty in conveying details such as primitive placement and articular surface selection through text and 2D images, we advocate that future ROM studies should prioritise providing 3D supplemental data. At minimum, this should include bone meshes, articular surfaces (either as separate meshes or as labelled regions on the bones), and the primitives used to define joint centres. As individual files (e.g. obj, ply) of bones, primitives, and articular surfaces need to share a common worldspace to be replicable, ideally the reference pose should be provided as a single scene file. This file should be in an open format capable of retaining the relevant data such as *.USD (Universal Scene Descriptor). Whilst software specific formats can be acceptable, those tied to open‐source tools (e.g., *.blend for Blender) are far preferable to closed, proprietary formats (e.g., *.mb/*.ma for Maya), which may be inaccessible to other researchers or become incompatible over time.

## AUTHOR CONTRIBUTIONS

R.J.L: conceptualisation, data curation, formal analysis, investigation, methodology, visualisation, writing – original draft, writing – review and editing; A.J.: conceptualisation, investigation, methodology, software, supervision, writing – review and editing; B.W.G: conceptualisation, investigation, methodology, software, supervision, writing – review and editing; T.L.P.: data curation, investigation, writing – review and editing; P.L.F.: conceptualisation, data curation, formal analysis, funding acquisition, investigation, methodology, project administration, resources, software, supervision, visualisation, writing – review and editing. All authors gave final approval for publication and agreed to be held accountable for the work performed therein.

## Supporting information


**Data S1:** Cylinder parameters and translations ranges.

Supporting information.

## Data Availability

The data that support the findings of this study are openly available in Figshare at https://doi.org/10.6084/m9.figshare.31746670.

## References

[joa70179-bib-0001] Anderst, W.J. , Vaidya, R. & Tashman, S. (2008) A technique to measure three‐dimensional in vivo rotation of fused and adjacent lumbar vertebrae. The Spine Journal, 8(6), 991–997. Available from: 10.1016/j.spinee.2007.07.390 17919983

[joa70179-bib-0002] Balolia, K.L. & Massey, J.S. (2021) How does scanner choice and 3D model resolution affect data accuracy? Journal of Anatomy, 238(3), 679–692. Available from: 10.1111/joa.13343 PMC7855060 33146411

[joa70179-bib-0003] Bishop, P.J. , Brocklehurst, R.J. & Pierce, S.E. (2022) Intelligent sampling of high‐dimensional joint mobility space for analysis of articular function. Methods in Ecology and Evolution, 14(2), 569–582. Available from: 10.1111/2041-210x.14016

[joa70179-bib-0004] Bishop, P.J. , Cuff, A.R. & Hutchinson, J.R. (2021) How to build a dinosaur: musculoskeletal modeling and simulation of locomotor biomechanics in extinct animals. Paleobiology, 47(1), 1–38. Available from: 10.1017/pab.2020.46

[joa70179-bib-0005] Blender Online Community . (2025) “Blender ‐ a 3D modelling and rendering package.”. https://www.blender.org

[joa70179-bib-0006] Bonnan, M.F. , Sandrik, J.L. , Nishiwaki, T. , Wilhite, D.R. , Elsey, R.M. & Vittore, C. (2010) Calcified cartilage shape in archosaur long bones reflects overlying joint shape in stress‐bearing elements: implications for nonavian dinosaur locomotion. The Anatomical Record, 293(12), 2044–2055. Available from: 10.1002/ar.21266 21046673

[joa70179-bib-0007] Brocklehurst, R.J. , Fahn‐Lai, L. , Biewener, A. & Pierce, S.E. (2025) Relationship between joint shape and function as revealed through ex vivo XROMM. Journal of Experimental Biology, 228(10), jeb249261. Available from: 10.1242/jeb.249261 40181760

[joa70179-bib-0008] Brocklehurst, R.J. , Fahn‐Lai, P. , Regnault, S. & Pierce, S.E. (2022) Musculoskeletal modeling of sprawling and parasagittal forelimbs provides insight into synapsid postural transition. iScience, 25(1), 103578. Available from: 10.1016/j.isci.2021.103578 PMC10441569 37609446

[joa70179-bib-0009] Chiang, C.‐Y. , Chen, K.‐H. , Liu, K.‐C. , Hsu, S.J.‐P. & Chan, C.‐T. (2017) Data collection and analysis using wearable sensors for monitoring knee range of motion after Total knee arthroplasty. Sensors, 17(2), 418. Available from: 10.3390/s17020418 PMC5336055 28241434

[joa70179-bib-0010] Comet Technologies Canada Inc . (2025) “Dragonfly 3D World.”. https://dragonfly.comet.tech/

[joa70179-bib-0011] Costa, F.R. , Rocha‐Barbosa, O. & Kellner, A.W.A. (2014) A biomechanical approach on the optimal stance of Anhanguera piscator (Pterodactyloidea) and its implications for pterosaur gait on land. Historical Biology, 26(5), 582–590. Available from: 10.1080/08912963.2013.807253

[joa70179-bib-0012] Costa, V. , Ramírez, Ó. , Otero, A. , Muñoz‐García, D. , Uribarri, S. & Raya, R. (2020) Validity and reliability of inertial sensors for elbow and wrist range of motion assessment. PeerJ, 8, e9687. Available from: 10.7717/peerj.9687 PMC7427560 32864213

[joa70179-bib-0013] Demuth, O.E. , Hutchinson, J.R. , La Barbera, V. , Warner, S.E. & Field, D.J. (2025) Soft tissue constraints on joint mobility in the avian shoulder. Journal of Experimental Biology, 228(21), jeb250952. Available from: 10.1242/jeb.250952 PMC12669841 40995780

[joa70179-bib-0014] Demuth, O.E. , Rayfield, E.J. & Hutchinson, J.R. (2020) 3D hindlimb joint mobility of the stem‐archosaur Euparkeria capensis with implications for postural evolution within Archosauria. Scientific Reports, 10(1), 15357. Available from: 10.1038/s41598-020-70175-y PMC7506000 32958770

[joa70179-bib-0015] Díez Díaz, V. , van Bijlert, P.A. , Sellers, W.I. , Wedel, M.J. & Schwarz, D. (2025) Centres of rotation and osteological constraints on caudal ranges of motion in the sauropod dinosaur Giraffatitan brancai. Royal Society Open Science, 12(8), 250851. Available from: 10.1098/rsos.250851 PMC12345363 40809361

[joa70179-bib-0016] Fahn‐Lai, P. , Biewener, A.A. & Pierce, S.E. (2018) Three‐dimensional mobility and muscle attachments in the pectoral limb of the Triassic cynodont Massetognathus pascuali (Romer, 1967). Journal of Anatomy, 232(3), 383–406. Available from: 10.1111/joa.12766 PMC5807948 29392730

[joa70179-bib-0017] Falkingham, P.L. (2016) Applying objective methods to subjective track outlines. In: Dinosaur tracks: the next steps. Bloomington: Indiana University Press, pp. 72–81.

[joa70179-bib-0018] Falkingham, P.L. & Gatesy, S.M. (2014) The birth of a dinosaur footprint: subsurface 3D motion reconstruction and discrete element simulation reveal track ontogeny. Proceedings of the National Academy of Sciences, 111(51), 18279–18284. Available from: 10.1073/pnas.1416252111 PMC4280635 25489092

[joa70179-bib-0019] Gatesy, S.M. , Bäker, M. & Hutchinson, J.R. (2009) Constraint‐based exclusion of limb poses for reconstructing theropod dinosaur locomotion. Journal of Vertebrate Paleontology, 29(2), 535–544. Available from: 10.1671/039.029.0213

[joa70179-bib-0020] Gatesy, S.M. , Manafzadeh, A.R. , Bishop, P.J. , Turner, M.L. , Kambic, R.E. , Cuff, A.R. et al. (2022) A proposed standard for quantifying 3‐D hindlimb joint poses in living and extinct archosaurs. Journal of Anatomy, 241(1), 101–118. Available from: 10.1111/joa.13635 PMC9178381 35118654

[joa70179-bib-0021] Griffin, B. , Martin‐Silverstone, E. , Demuth, O. , Pêgas, R. , Palmer, C. & Rayfield, E. (2022) Constraining pterosaur launch: range of motion in the pectoral and pelvic girdles of a medium‐sized ornithocheiraean pterosaur. Biological Journal of the Linnean Society, 137(2), 250–266. Available from: 10.1093/biolinnean/blac063

[joa70179-bib-0022] Hancock, G.E. , Hepworth, T. & Wembridge, K. (2018) Accuracy and reliability of knee goniometry methods. Journal of Experimental Orthopaedics, 5(1), 46. Available from: 10.1186/s40634-018-0161-5 PMC6195503 30341552

[joa70179-bib-0023] Herbst, E.C. , Eberhard, E.A. , Richards, C.T. & Hutchinson, J.R. (2022) In vivo and ex vivo range of motion in the fire salamander Salamandra salamandra. Journal of Anatomy, 241(4), 1066–1082. Available from: 10.1111/joa.13738 PMC9482696 35986620

[joa70179-bib-0024] Hicks, J.L. , Uchida, T.K. , Seth, A. , Rajagopal, A. & Delp, S.L. (2015) Is my model good enough? Best practices for verification and validation of musculoskeletal models and simulations of movement. Journal of Biomechanical Engineering, 137(2), 0209051–02090524. Available from: 10.1115/1.4029304 PMC4321112 25474098

[joa70179-bib-0025] Holliday, C.M. , Ridgely, R.C. , Sedlmayr, J.C. & Witmer, L.M. (2010) Cartilaginous epiphyses in extant archosaurs and their implications for reconstructing limb function in dinosaurs. PLoS One, 5(9), e13120. Available from: 10.1371/journal.pone.0013120 PMC2948032 20927347

[joa70179-bib-0026] Hutson, J.D. & Hutson, K.N. (2012) A test of the validity of range of motion studies of fossil archosaur elbow mobility using repeated‐measures analysis and the extant phylogenetic bracket. Journal of Experimental Biology, 215(12), 2030–2038. Available from: 10.1242/jeb.069567 22623191

[joa70179-bib-0027] Jaeger, G.H. , Marcellin‐Little, D.J. , DePuy, V. & Lascelles, B.D.X. (2007) Validity of goniometric joint measurements in cats. American Journal of Veterinary Research, 68(8), 822–826. Available from: 10.2460/ajvr.68.8.822 17669021

[joa70179-bib-0028] Jones, K.E. , Brocklehurst, R.J. & Pierce, S.E. (2021) AutoBend: an automated approach for estimating intervertebral joint function from bone‐only digital models. Integrative Organismal Biology, 3(1), obab026. Available from: 10.1093/iob/obab026 PMC8514422 34661062

[joa70179-bib-0029] Kambic, R.E. , Roberts, T.J. & Gatesy, S.M. (2014) Long‐axis rotation: a missing degree of freedom in avian bipedal locomotion. Journal of Experimental Biology, 217(15), jeb.101428. Available from: 10.1242/jeb.101428 24855675

[joa70179-bib-0030] Kambic, R.E. , Roberts, T.J. & Gatesy, S.M. (2017) 3‐D range of motion envelopes reveal interacting degrees of freedom in avian hind limb joints. Journal of Anatomy, 231(6), 906–920. Available from: 10.1111/joa.12680 PMC5696129 28833095

[joa70179-bib-0031] Kim, S.E. , Jones, S.C. , Lewis, D.D. , Banks, S.A. , Conrad, B.P. , Tremolada, G. et al. (2015) In‐vivo three‐dimensional knee kinematics during daily activities in dogs. Journal of Orthopaedic Research, 33(11), 1603–1610. Available from: 10.1002/jor.22927 25982776

[joa70179-bib-0032] Mallison, H. (2010a) CAD assessment of the posture and range of motion of Kentrosaurus aethiopicus Hennig 1915. Swiss Journal of Geosciences, 103(2), 211–233. Available from: 10.1007/s00015-010-0024-2

[joa70179-bib-0033] Mallison, H. (2010b) The digital *Plateosaurus* II: an assessment of the range of motion of the limbs and vertebral column and of previous reconstructions using a digital skeletal mount. Acta Palaeontologica Polonica, 55(3), 433–458. Available from: 10.4202/app.2009.0075

[joa70179-bib-0034] Manafzadeh, A.R. & Gatesy, S.M. (2020) A coordinate‐system‐independent method for comparing joint rotational mobilities. Journal of Experimental Biology, 223(18), jeb227108. Available from: 10.1242/jeb.227108 32747453

[joa70179-bib-0035] Manafzadeh, A.R. & Gatesy, S.M. (2021) Paleobiological reconstructions of articular function require all six degrees of freedom. Journal of Anatomy, 239(6), 1516–1524. Available from: 10.1111/joa.13513 PMC8602027 34275132

[joa70179-bib-0036] Manafzadeh, A.R. & Gatesy, S.M. (2022) Advances and challenges in Paleobiological reconstructions of joint mobility. Integrative and Comparative Biology, 62(5), 1369–1376. Available from: 10.1093/icb/icac008 35289839

[joa70179-bib-0037] Manafzadeh, A.R. , Gatesy, S.M. & Bhullar, B.‐A.S. (2024) Articular surface interactions distinguish dinosaurian locomotor joint poses. Nature Communications, 15(1), 854. Available from: 10.1038/s41467-024-44832-z PMC10873393 38365765

[joa70179-bib-0038] Manafzadeh, A.R. & Padian, K. (2018) ROM mapping of ligamentous constraints on avian hip mobility: implications for extinct ornithodirans. Proceedings of the Royal Society B: Biological Sciences, 285(1879), 20180727. Available from: 10.1098/rspb.2018.0727 PMC5998106 29794053

[joa70179-bib-0039] MathWorks Inc . (2025) “MATLAB R2025a.”. https://uk.mathworks.com/products/matlab.html

[joa70179-bib-0040] Molnar, J.L. , Hutchinson, J.R. , Diogo, R. , Clack, J.A. & Pierce, S.E. (2021) Evolution of forelimb musculoskeletal function across the fish‐to‐tetrapod transition. Science Advances, 7(4), eabd7457. Available from: 10.1126/sciadv.abd7457 PMC10964964 33523947

[joa70179-bib-0041] Oakes, R. , Hill Chase, M. , Siddall, M. & Sessa, J. (2020) Testing the impact of two key scan parameters on the quality and repeatability of measurements from CT scan data. Palaeontologia Electronica, 23(1), a07. Available from: 10.26879/942

[joa70179-bib-0042] Peebles, A.T. , Carroll, M.M. , Socha, J.J. , Schmitt, D. & Queen, R.M. (2021) Validity of using automated two‐dimensional video analysis to measure continuous sagittal plane running kinematics. Annals of Biomedical Engineering, 49(1), 455–468. Available from: 10.1007/s10439-020-02569-y 32705424

[joa70179-bib-0043] Pierce, S.E. , Clack, J.A. & Hutchinson, J.R. (2012) Three‐dimensional limb joint mobility in the early tetrapod Ichthyostega. Nature, 486(7404), 523–526. Available from: 10.1038/nature11124 22722854

[joa70179-bib-0044] R Core Team . (2025) “R: A Language and Environment for Statistical Computing.”. https://www.r‐project.org/

[joa70179-bib-0045] Regnault, S. & Pierce, S.E. (2018) Pectoral girdle and forelimb musculoskeletal function in the echidna (Tachyglossus aculeatus): insights into mammalian locomotor evolution. Royal Society Open Science, 5(11), 181400. Available from: 10.1098/rsos.181400 PMC6281926 30564424

[joa70179-bib-0046] Richards, H.L. , Bishop, P.J. , Hocking, D.P. , Adams, J.W. & Evans, A.R. (2021) Low elbow mobility indicates unique forelimb posture and function in a giant extinct marsupial. Journal of Anatomy, 238(6), 1425–1441. Available from: 10.1111/joa.13389 PMC8128769 33533053

[joa70179-bib-0047] Scheidt, A. , Renk, A.C.E. & Nyakatura, J.A. (2026) Scaling of internal joint distance in the elbow of small‐ to medium‐sized mammals: implications for range of motion analyses. Journal of Anatomy, 248, 950–971. Available from: 10.1111/joa.70116 PMC13148638 41652842

[joa70179-bib-0048] Senter, P. & Robins, J.H. (2005) Range of motion in the forelimb of the theropod dinosaur *Acrocanthosaurus atokensis*, and implications for predatory behaviour. Journal of Zoology, 266(3), 307–318. Available from: 10.1017/S0952836905006989

[joa70179-bib-0049] Senter, P. & Sullivan, C. (2019) Forelimbs of the theropod dinosaur Dilophosaurus wetherilli: range of motion, influence of paleopathology and soft tissues, and description of a distal carpal bone. Palaeontologia Electronica, 22, 1–19. Available from: 10.26879/900

[joa70179-bib-0050] Voegele, K.K. , Bonnan, M.F. , Siegler, S. , Langel, C.R. & Lacovara, K.J. (2022) Constraining morphologies of soft tissues in extinct vertebrates using multibody dynamic simulations: a case study on articular cartilage of the sauropod Dreadnoughtus. Frontiers in Earth Science, 10, 786247. Available from: 10.3389/feart.2022.786247

[joa70179-bib-0051] White, M.A. , Bell, P.R. , Cook, A.G. , Barnes, D.G. , Tischler, T.R. , Bassam, B.J. et al. (2015) Forearm range of motion in Australovenator wintonensis (*Theropoda*, *Megaraptoridae*). PLoS One, 10(9), e0137709. Available from: 10.1371/journal.pone.0137709 PMC4569425 26368529

[joa70179-bib-0052] Wilke, H.J. , Herkommer, A. , Werner, K. & Liebsch, C. (2017) In vitro analysis of the segmental flexibility of the thoracic spine. PLoS One, 12(5), e0177823. Available from: 10.1371/journal.pone.0177823 PMC5433776 28520819

[joa70179-bib-0053] Witmer, L.M. (1995) The extant phylogenetic bracket and the importance of reconstructing soft tissues in fossils. In: Thomason, J.J. (Ed.) Functional morphology in vertebrate paleontology. Cambridge: Cambridge University Press, pp. 19–33.

[joa70179-bib-0054] Wu, G. , Siegler, S. , Allard, P. , Kirtley, C. , Leardini, A. , Rosenbaum, D. et al. (2002) ISB recommendation on definitions of joint coordinate system of various joints for the reporting of human joint motion—part I: ankle, hip, and spine. Journal of Biomechanics, 35(4), 543–548. Available from: 10.1016/S0021-9290(01)00222-6 11934426

