# Supplementary material for: Digital range of motion analysis is sensitive to subjective steps in joint model construction
Source: J Anat. 2026 Jun 3;249(6):1327–44. doi: 10.1111/joa.70179 (PMC13398749; doi:10.1111/joa.70179)
Supplement: Supplementary file 1 [file JOA-249-1327-s001.pdf]

Digital range of motion analysis is sensitive to subjective steps in joint model construction

R.J. Lowes[1], A. Jannel[1], B.W. Griffin[1], T.L. Prescott[1], P.L. Falkingham[1]

[1]School of Biological and Environmental Sciences, Liverpool John Moores University, Liverpool, UK

Supplementary table 1: Manual rotation of each ACSf axis for SA2 when fitting a cylinder, plane and sphere to the distal articular surface of the proximal bone within the joint. Abbreviations: TMTP3, tarsometatarsophalangeal III.

**Joint**	**Proximal bone's distal primitive**	**ACSf manual rotation (°)**		
		**X**	**Y**	**Z**
TMTP3	Cylinder	0	0	0
	Plane	170	-50	-5
	Sphere	-10	-7	0
Ankle	Cylinder	0	0	0
	Plane	-86	-19	0
	Sphere	9	0	0

Supplementary table 2: Number of faces for each mesh and joint at the different resolutions tested in SA5. Abbreviations: TMTP3, tarsometatarsophalangeal III; TMT, tarsometatarsus; PIII-1, phalanx III-1.

Joint	Tested resolution	Mesh	Faces	Joint total faces	Faces/cm2
TMTP3	Full	TMT	220417	256170	9230.92
		PIII-1	35753		
	0.5x	TMT	110208	128084	4787.86
		PIII-1	17876		
	0.25x	TMT	55104	64042	2391.69
		PIII-1	8938		
	0.125x	TMT	27552	32020	1196.43
		PIII-1	4468		
	<1000 faces	TMT	8264	9604	358.40
		PIII-1	1340		
Ankle	Full	TBT	422466	642883	9416.41
		TMT	220417		
	0.5x	TBT	211232	321440	4708.12
		TMT	110208		
	0.25x	TBT	105616	160720	2353.95
		TMT	55104		
	0.125x	TBT	52808	80360	1176.84
		TMT	27552		
	<1000 faces	TBT	5808	9664	141.24
		TMT	3856		

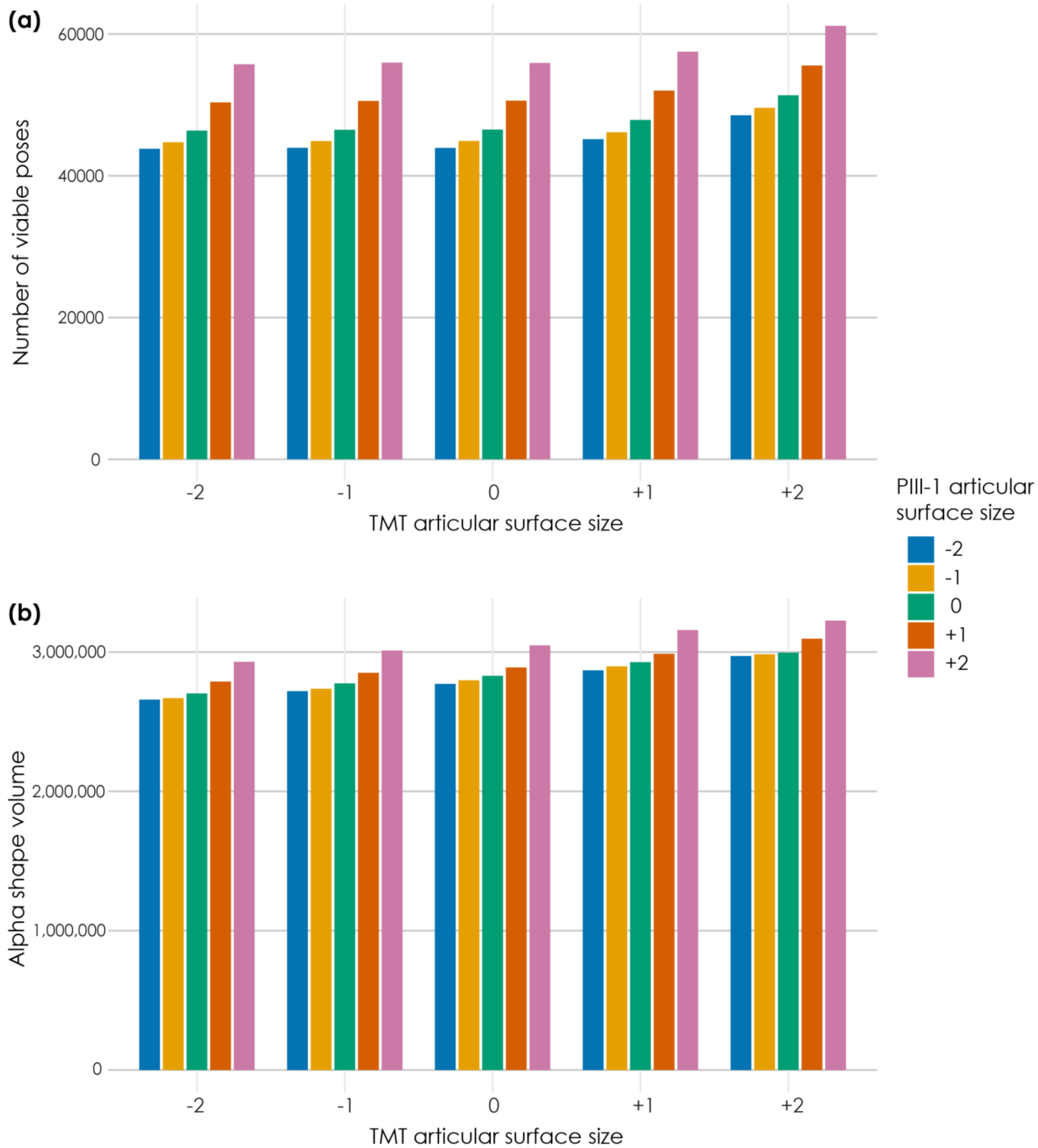

Supplementary fig.1: Number of viable poses (a) and alpha shape volume (b) for each trial of SA1 for the TMTP3 joint. Abbreviations: TMT, tarsometatarsus; PIII-1, phalanx III-1.

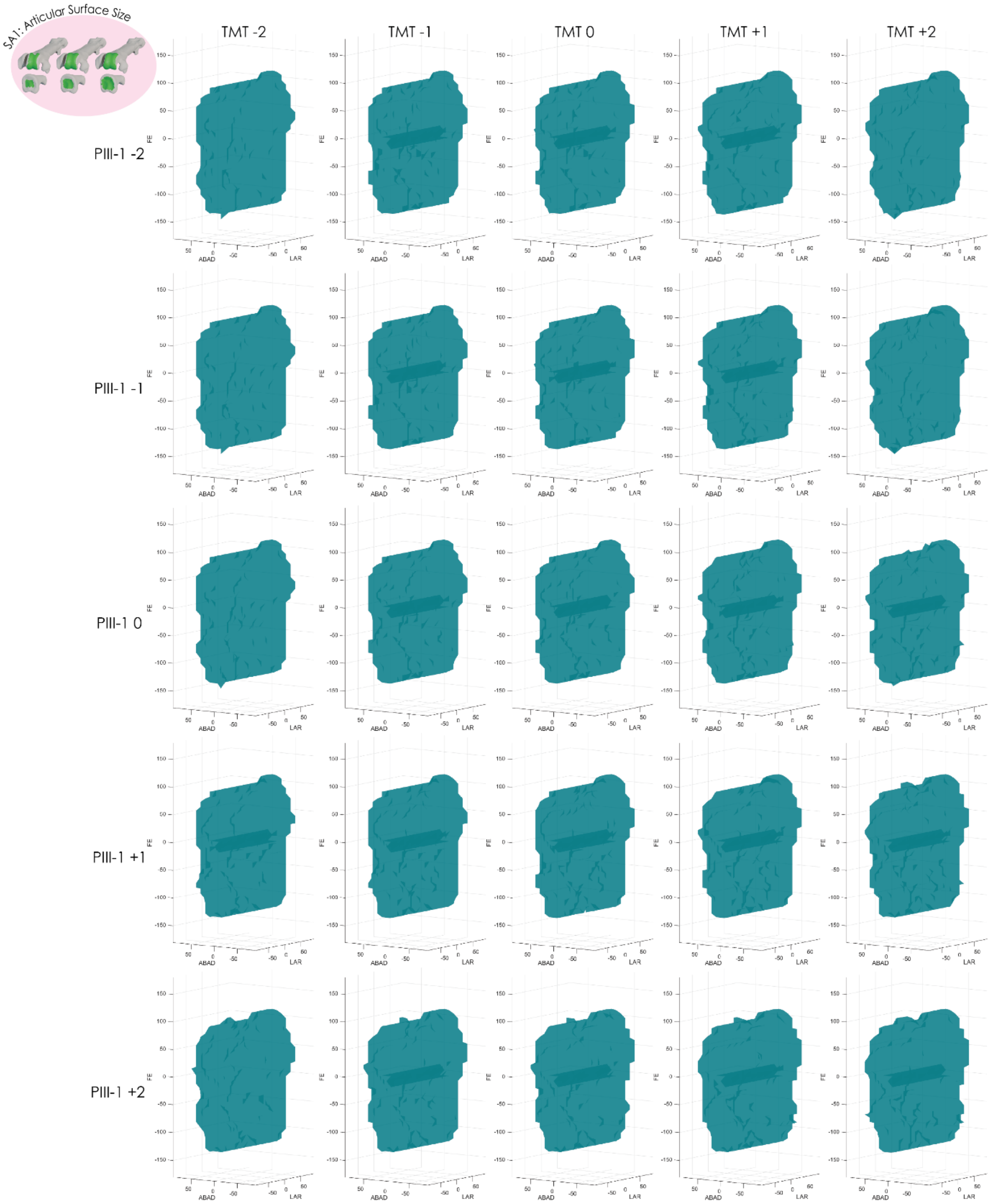

Supplementary fig.2: Cosine-corrected 3D range of motion maps for each trial of SA1 for the TMTP3 joint. Axes show flexion/extension (FE), abduction/adduction (ABAD) and long-axis rotation (LAR) angles in degrees at 10-degree resolution. Abbreviations: TMT, tarsometatarsus; PIII-1, phalanx III-1.

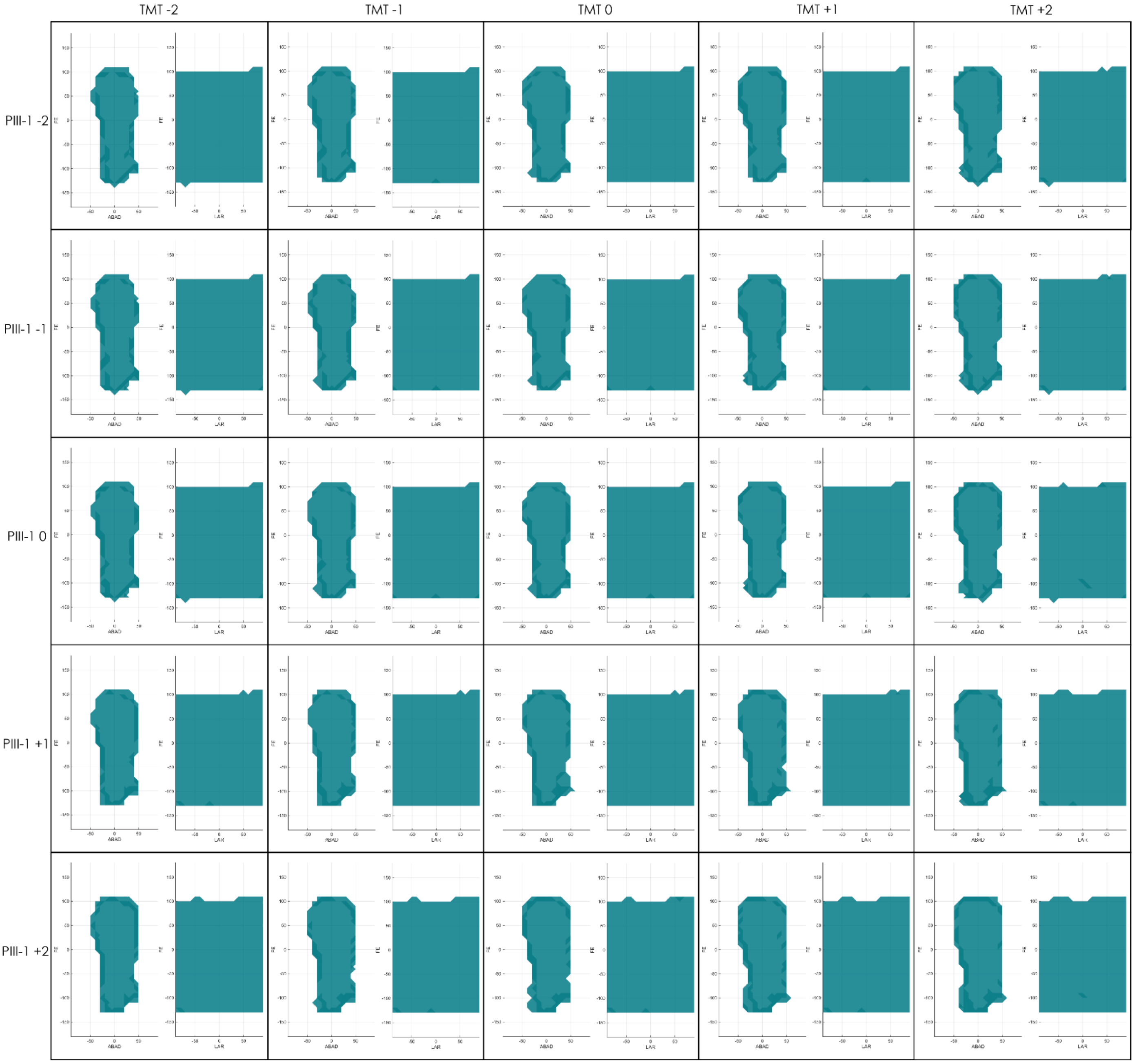

Supplementary fig.3: 2D range of motion maps for each trial of SA1 for the TMTP3 joint to show maximum rotational angles. Axes show flexion/extension (FE), abduction/adduction (ABAD) and long-axis rotation (LAR) angles in degrees at 10-degree resolution. Abbreviations: TMT, tarsometatarsus; PIII-1, phalanx III-1.

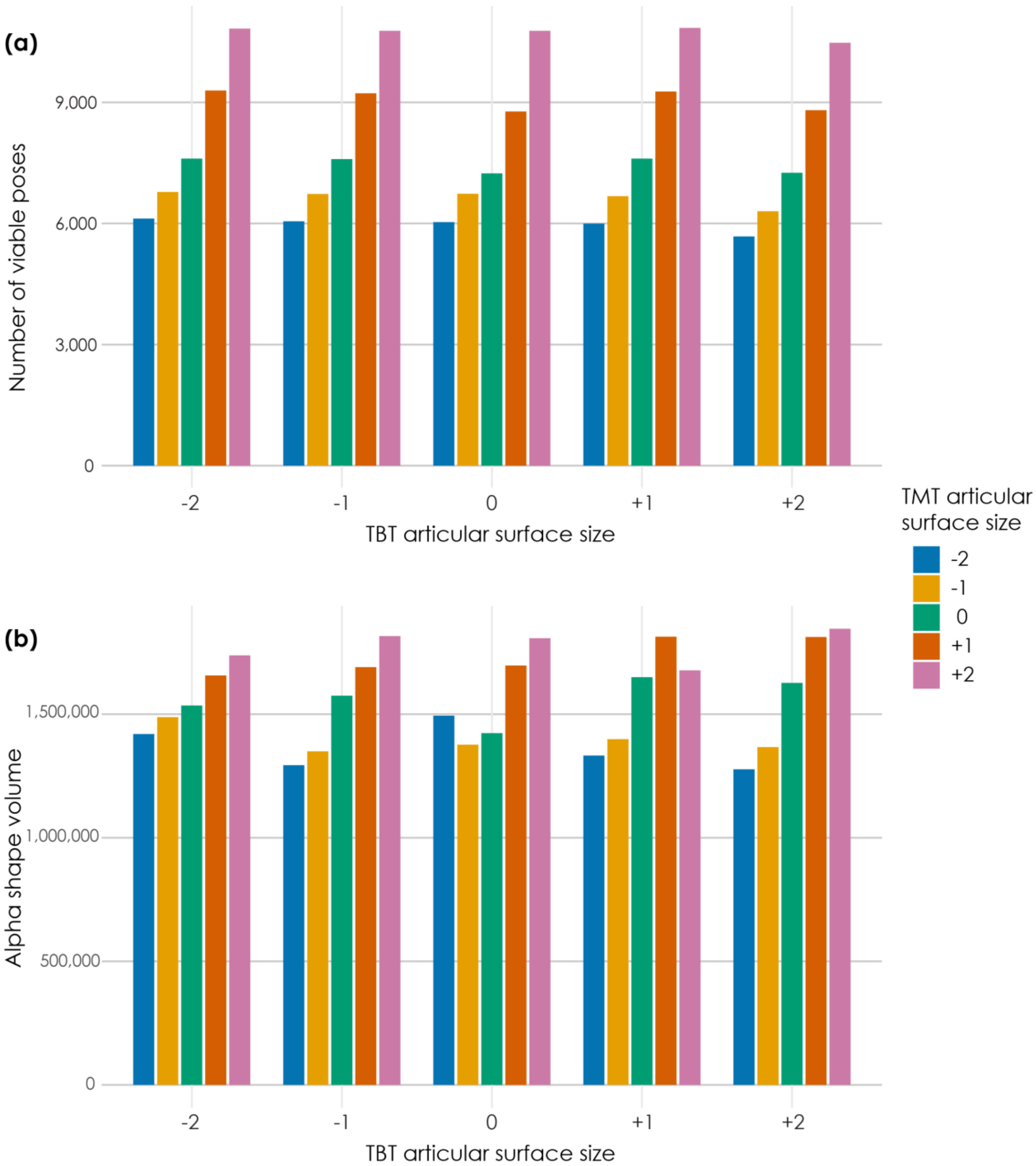

Supplementary fig.4: Number of viable poses (a) and alpha shape volume (b) for each trial of SA1 for the ankle joint. Abbreviations: TBT, tibiotarsus; TMT, tarsometatarsus.

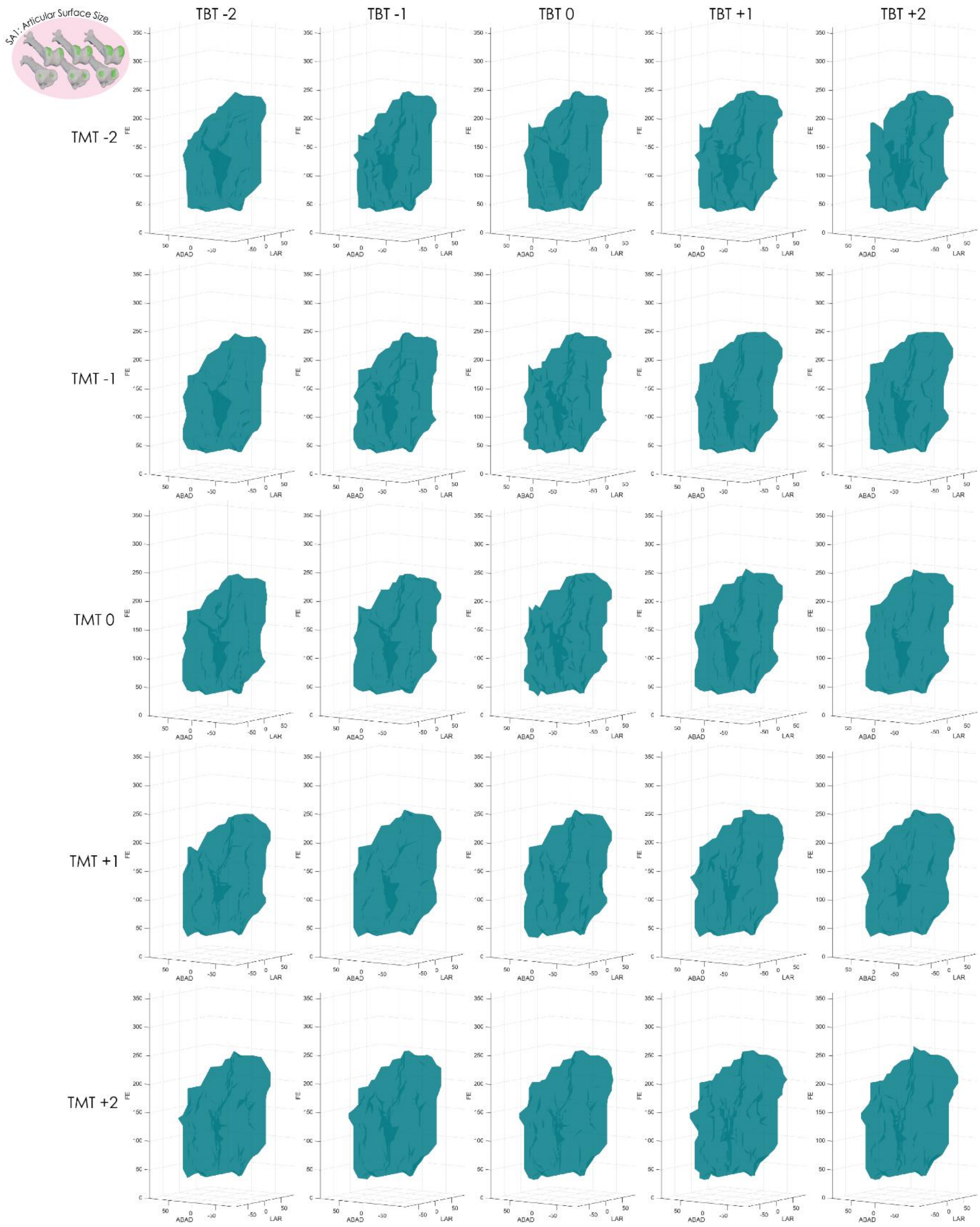

Supplementary fig.5: Cosine-corrected 3D range of motion maps for each trial of SA1 for the ankle joint. Axes show flexion/extension (FE), abduction/adduction (ABAD) and long-axis rotation (LAR) angles in degrees at 10-degree resolution. Abbreviations: TBT, tibiotarsus; TMT, tarsometatarsus.

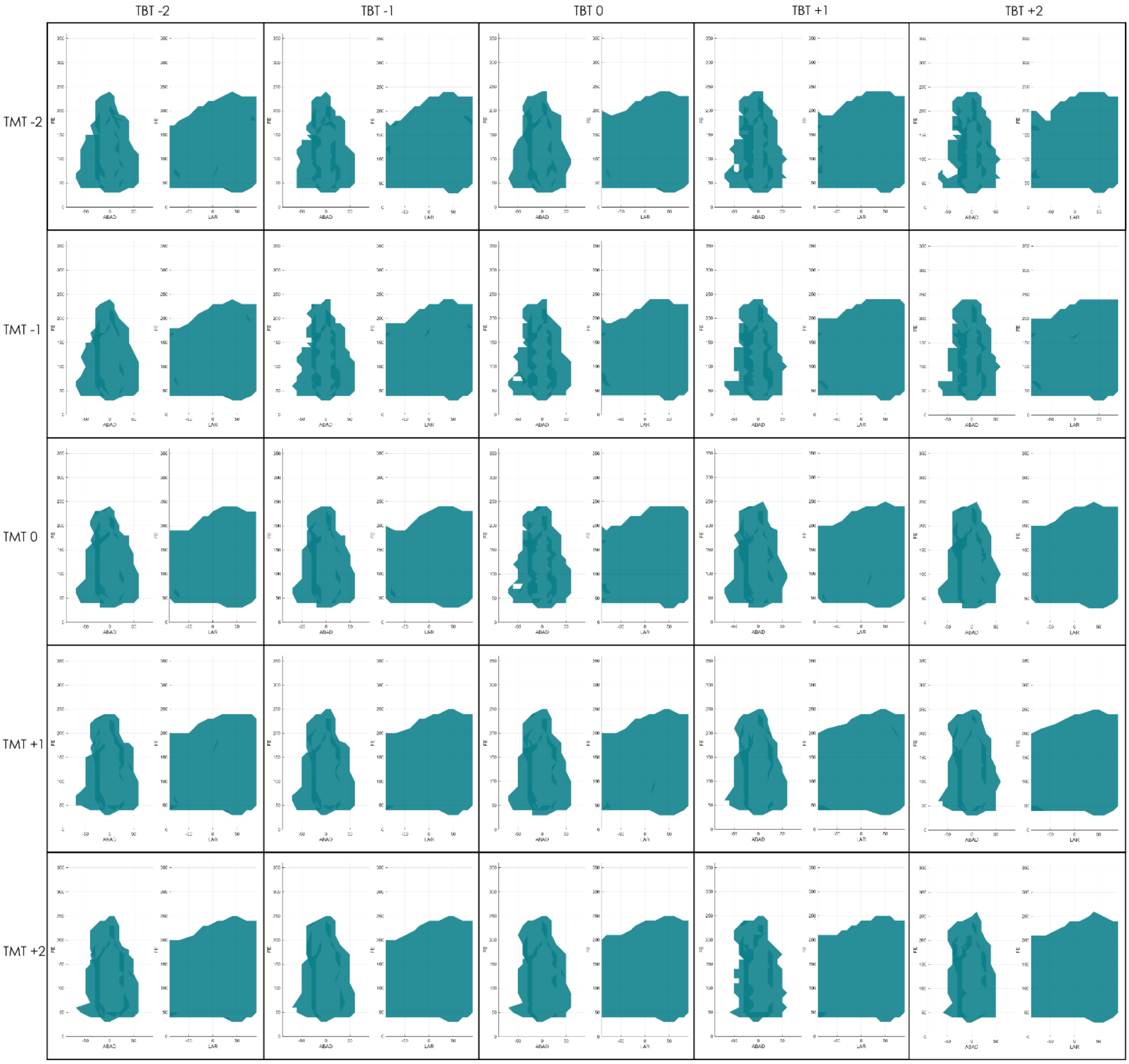

Supplementary fig.6: 2D range of motion maps for each trial of SA1 for the ankle joint to show maximum rotational angles. Axes show flexion/extension (FE), abduction/adduction (ABAD) and long-axis rotation (LAR) angles in degrees at 10-degree resolution. Abbreviations: TBT, tibiotarsus; TMT, tarsometatarsus.

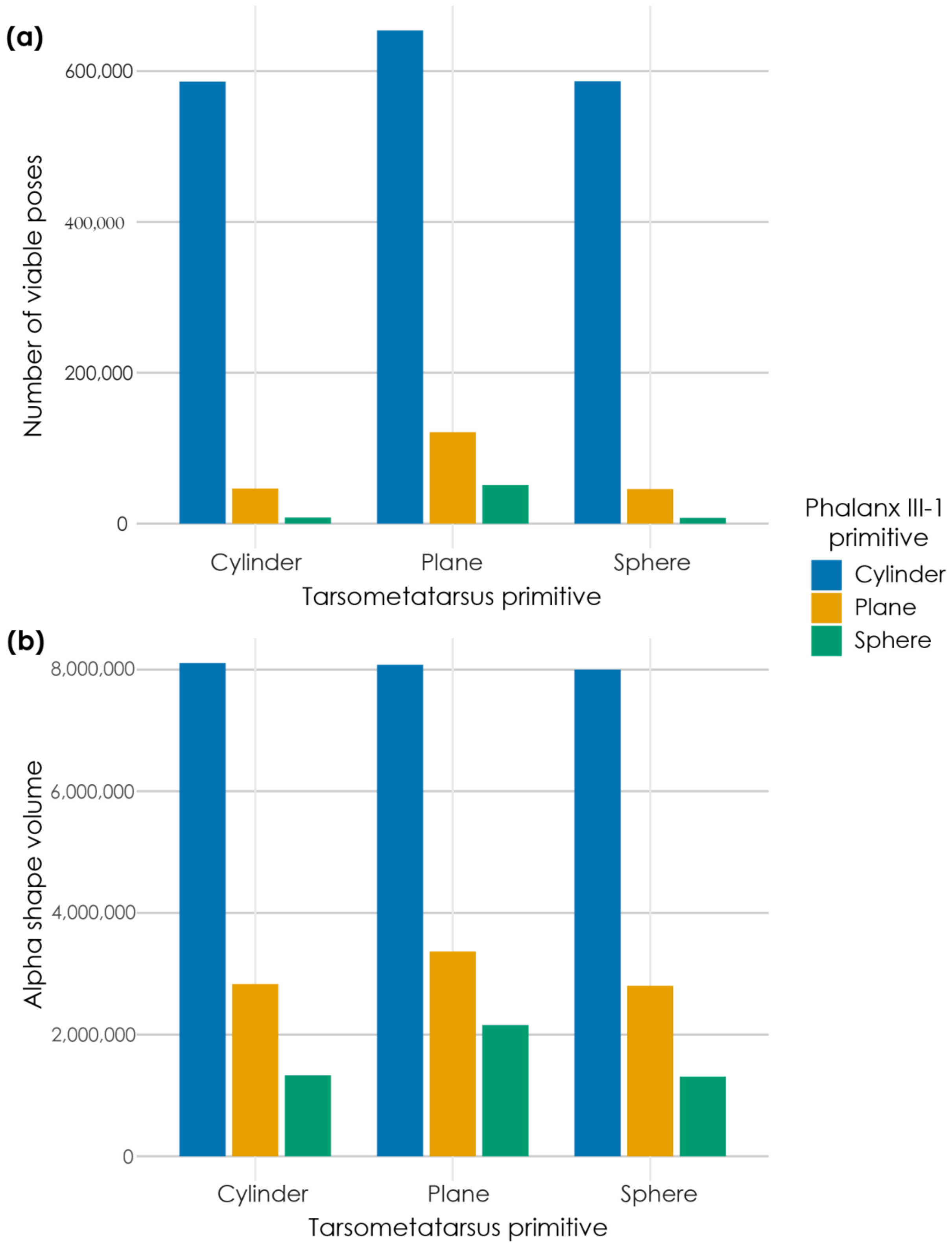

Supplementary fig.7: Number of viable poses (a) and alpha shape volume (b) for each trial of SA2 for the TMTP3 joint.

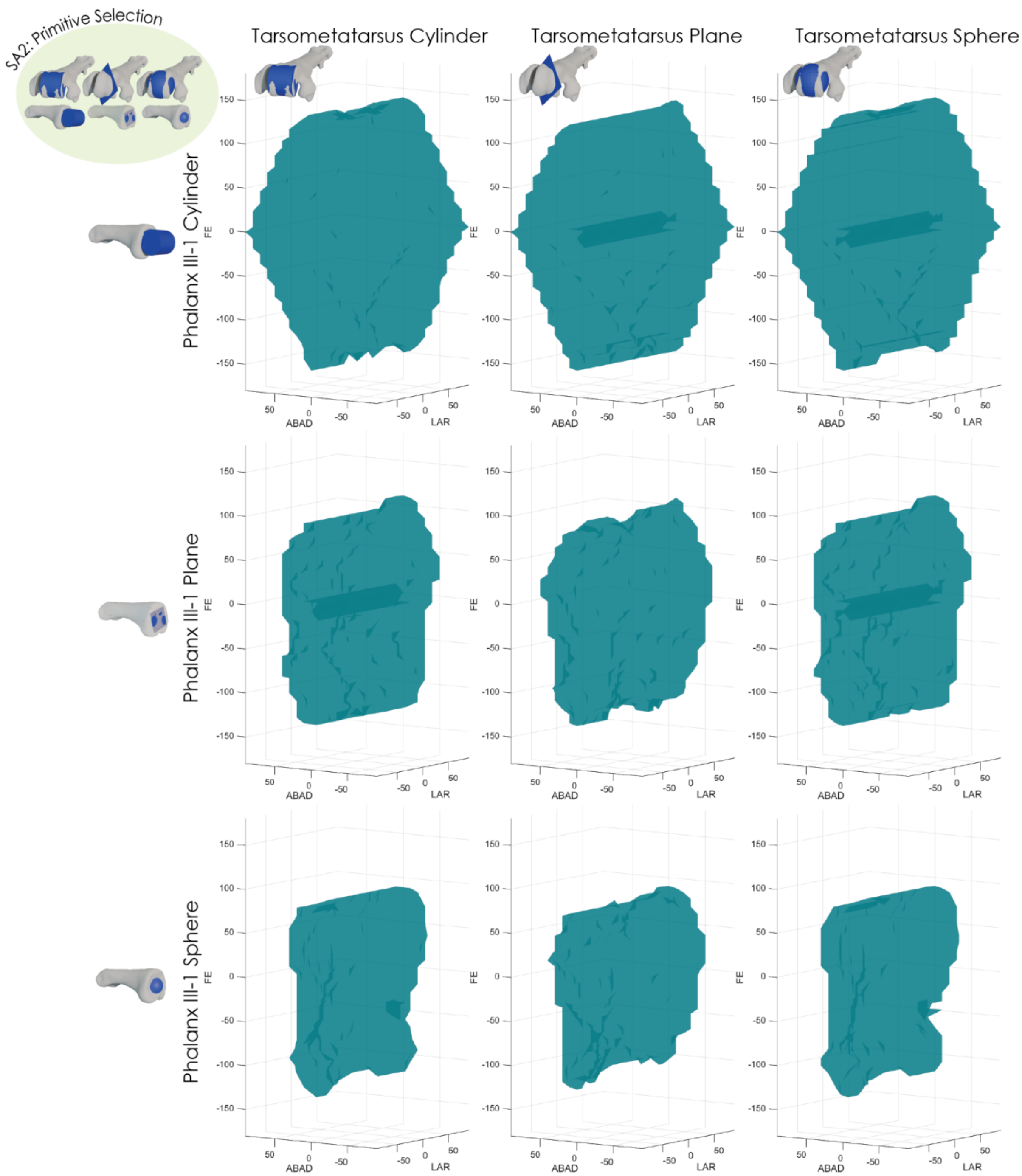

Supplementary fig.8: Cosine-corrected 3D range of motion maps for each trial of SA2 for the TMTP3 joint. Axes show flexion/extension (FE), abduction/adduction (ABAD) and long-axis rotation (LAR) angles in degrees at 10-degree resolution.

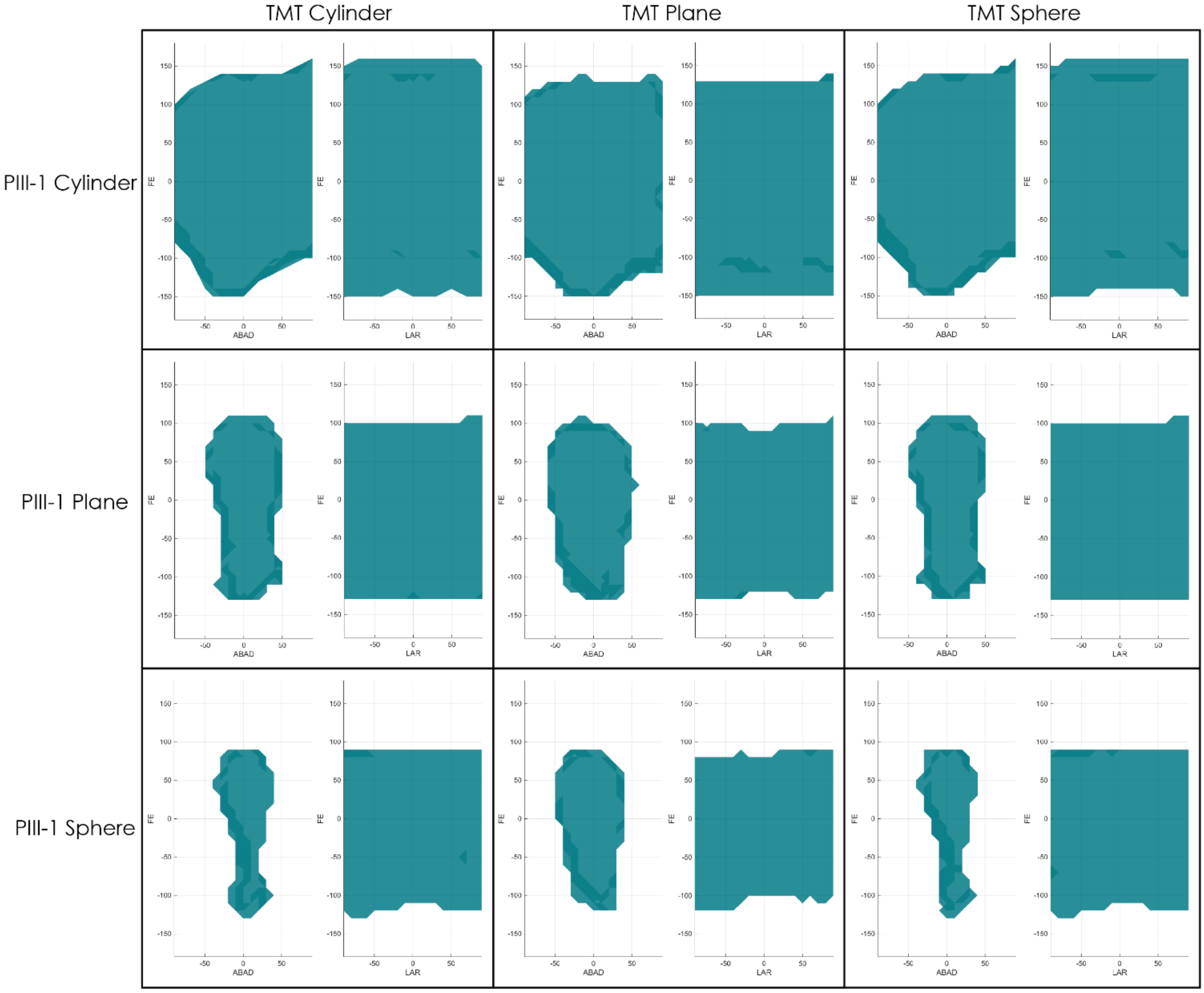

Supplementary fig.9: 2D range of motion maps for each trial of SA1 for the ankle joint to show maximum rotational angles. Axes show flexion/extension (FE), abduction/adduction (ABAD) and long-axis rotation (LAR) angles in degrees at 10-degree resolution. Abbreviations: TMT, tarsometatarsus, PIII-1, phalanx III-1.

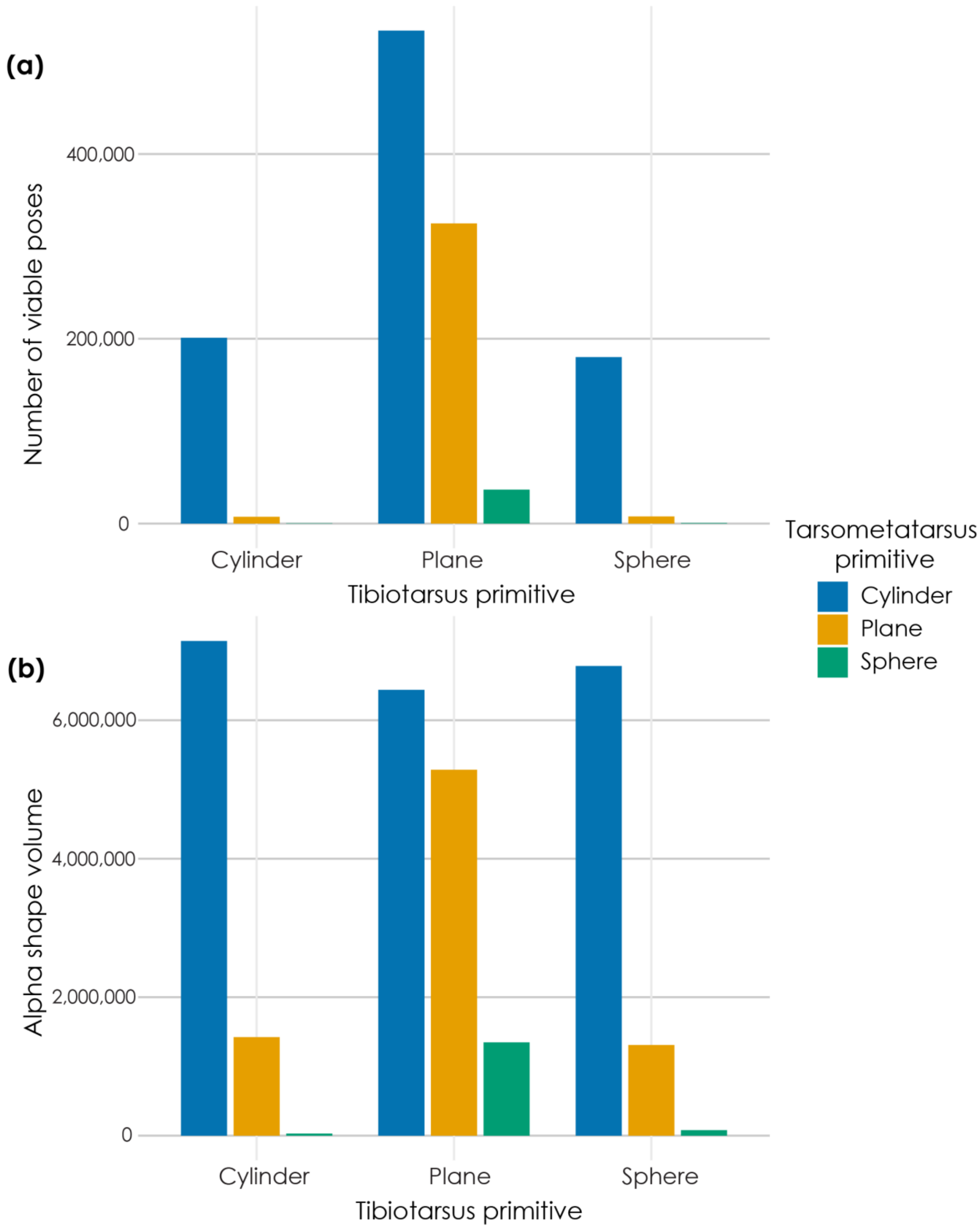

Supplementary fig.10: Number of viable poses (a) and alpha shape volume (b) for each trial of SA2 for the ankle joint.

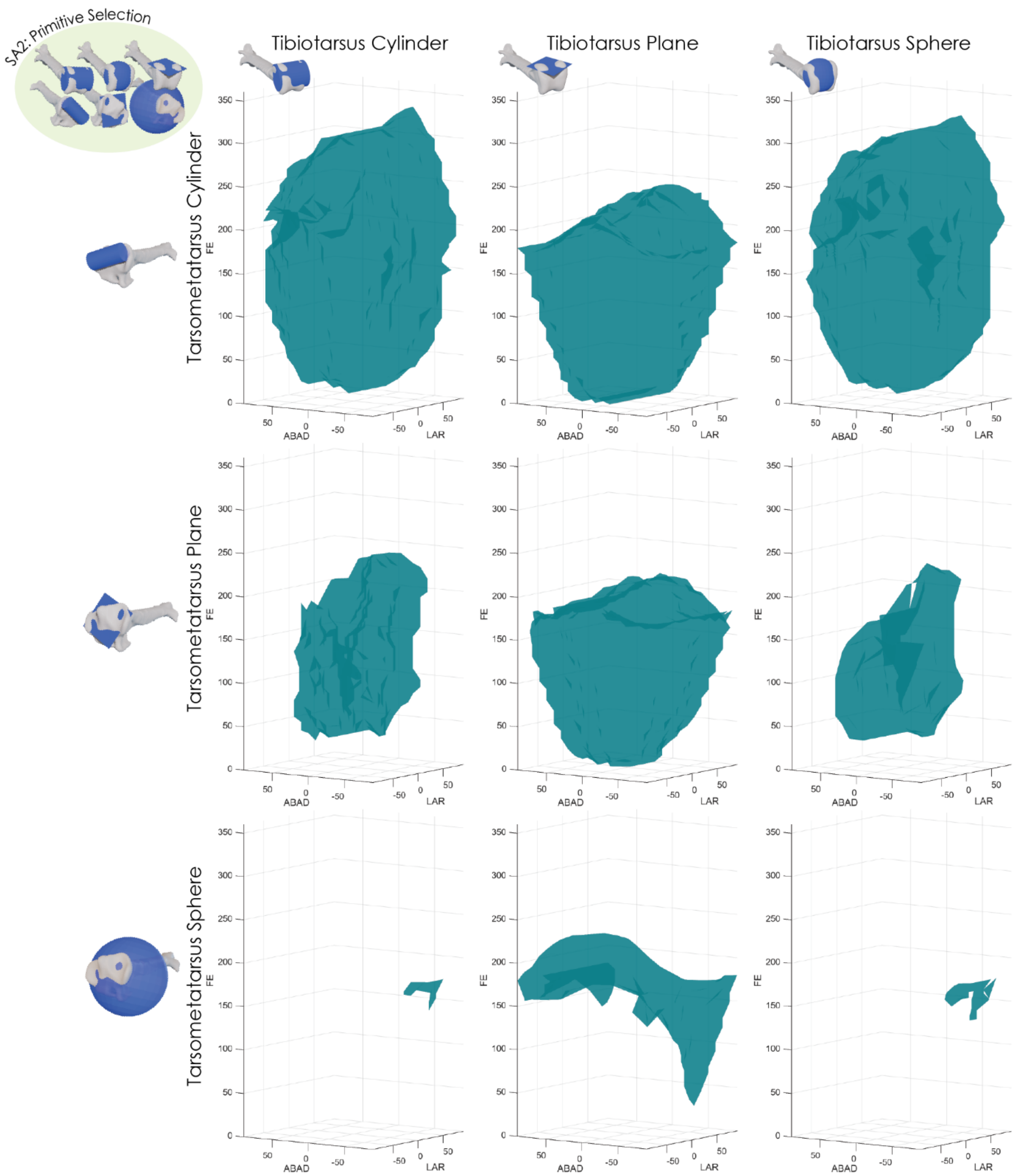

Supplementary fig.11: Cosine-corrected 3D range of motion maps for each trial of SA1 for the ankle joint. Axes show flexion/extension (FE), abduction/adduction (ABAD) and long-axis rotation (LAR) angles in degrees at 10-degree resolution.

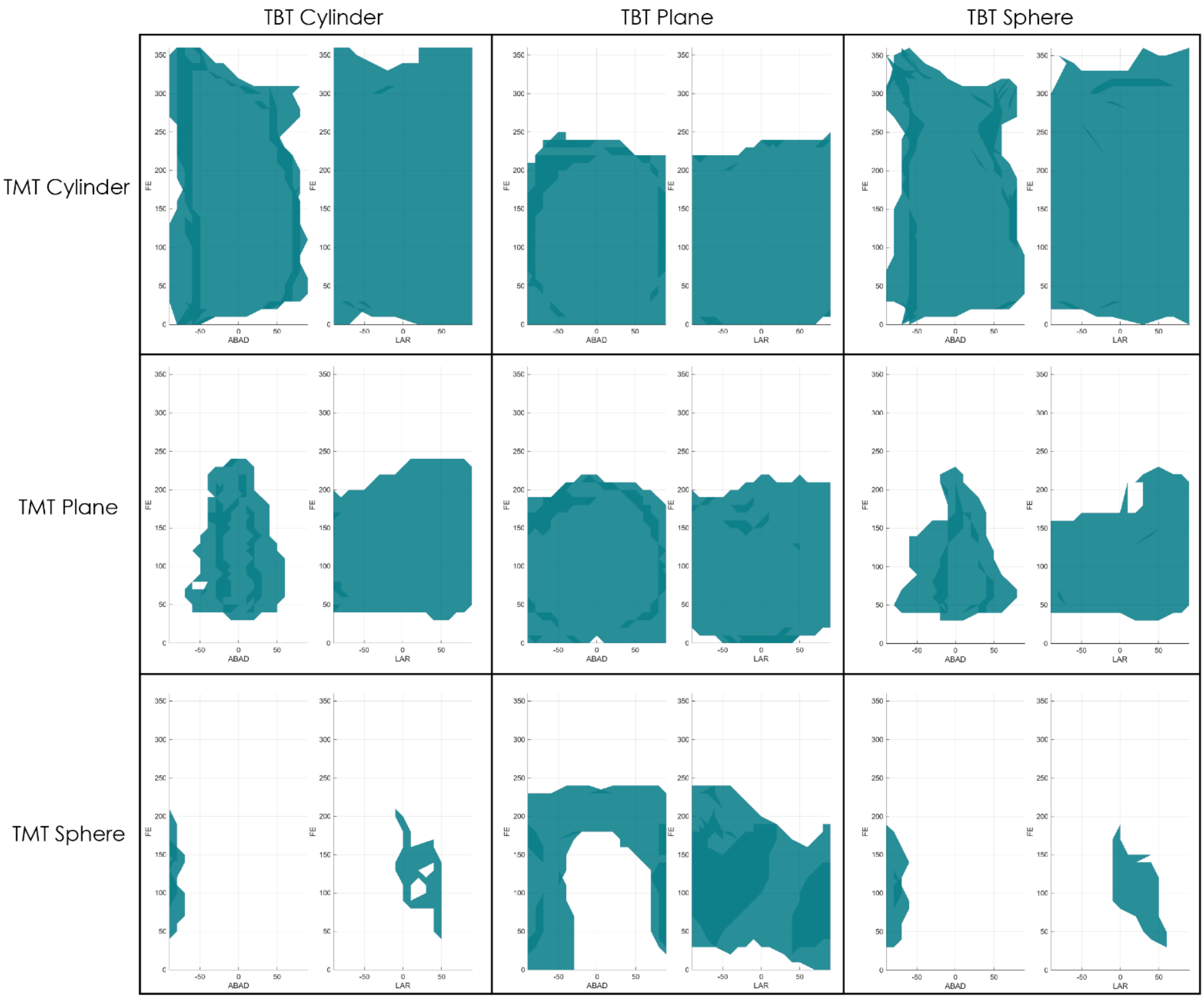

Supplementary fig.12: 2D range of motion maps for each trial of SA2 for the ankle joint to show maximum rotational angles. Axes show flexion/extension (FE), abduction/adduction (ABAD) and long-axis rotation (LAR) angles in degrees at 10-degree resolution. Abbreviations: TBT, tibiotarsus; TMT, tarsometatarsus.

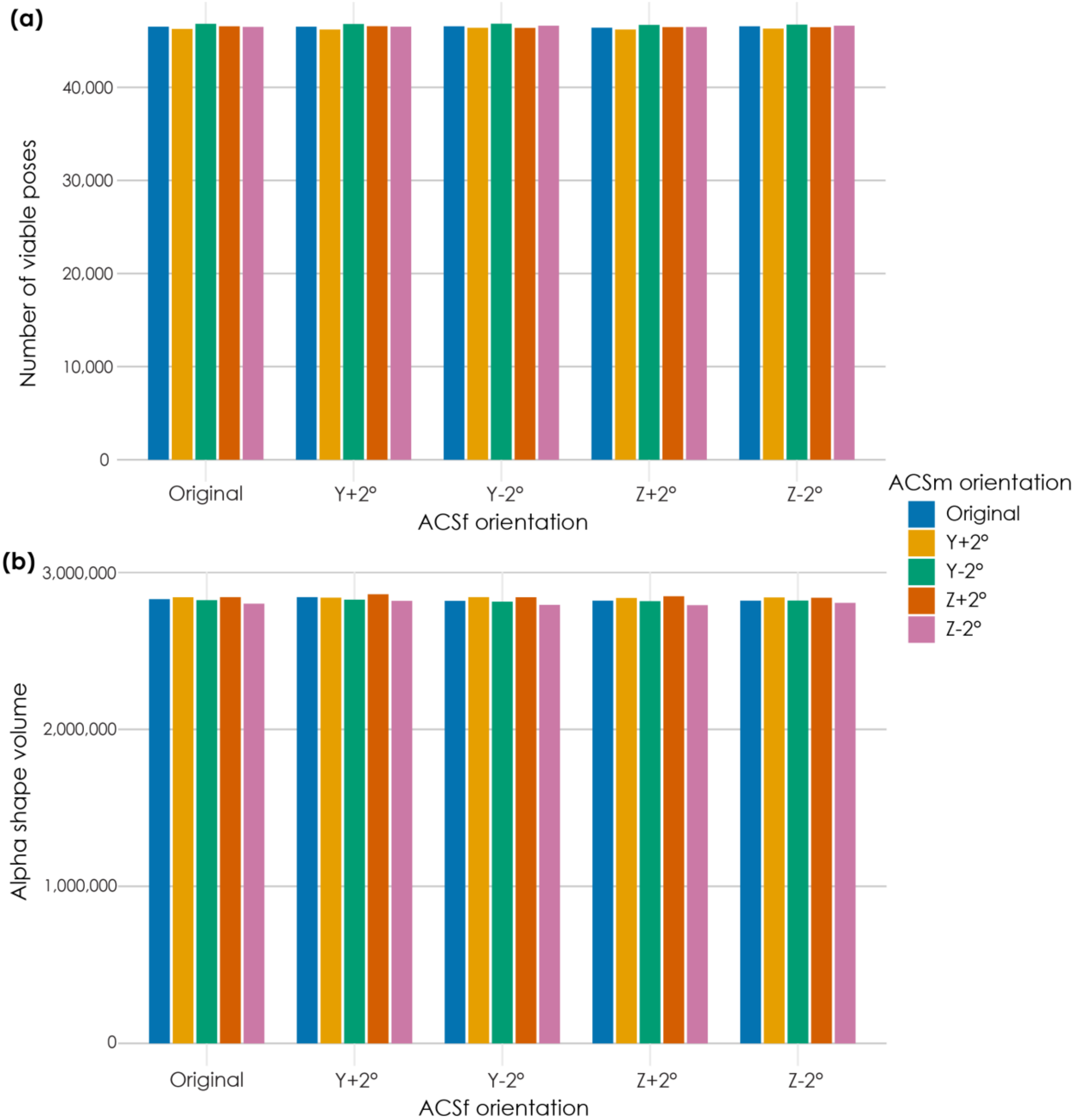

Supplementary fig.13: Number of viable poses (a) and alpha shape volume (b) for each trial of SA3 for the TMTP3 joint.

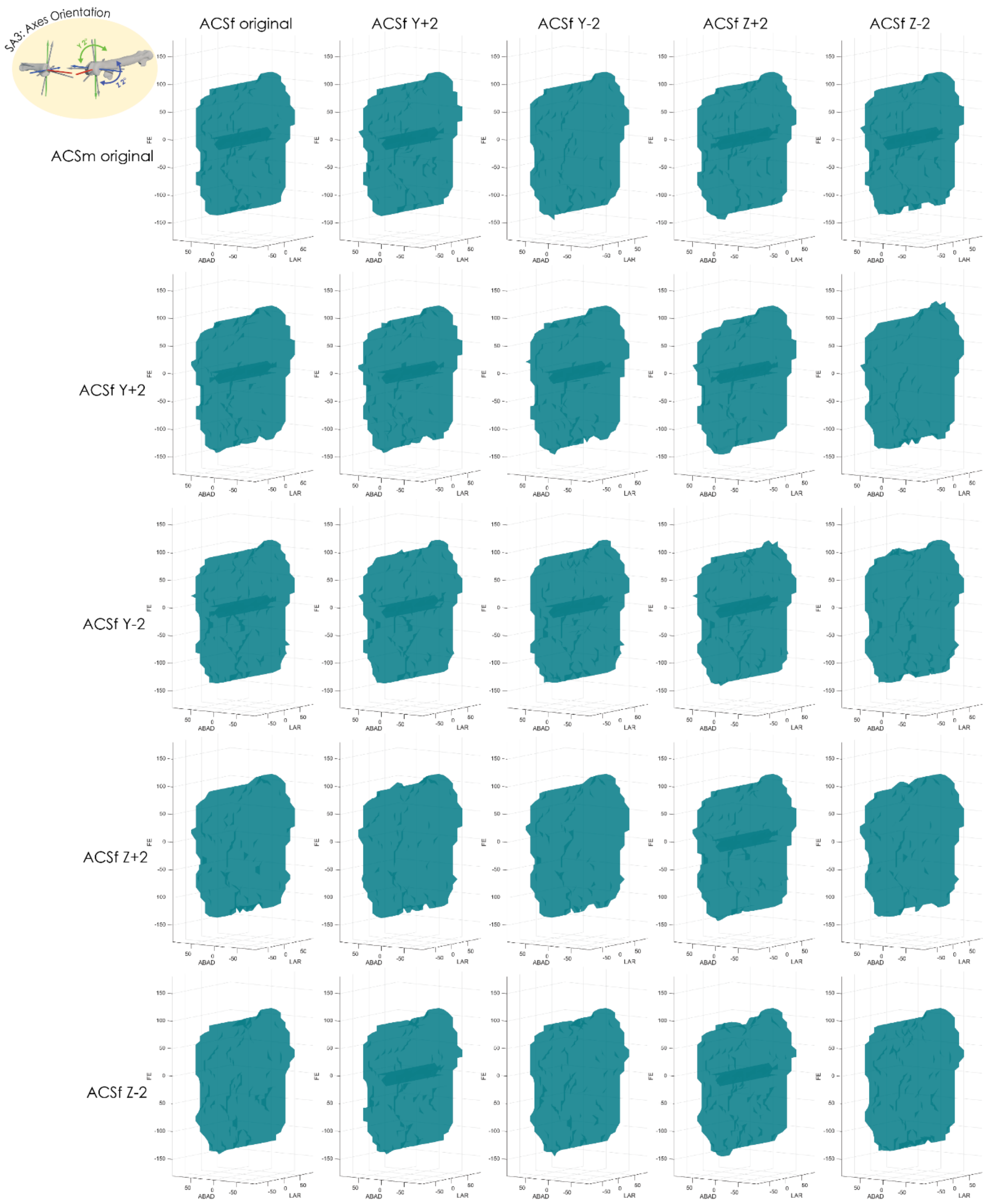

Supplementary fig.14: Cosine-corrected 3D range of motion maps for each trial of SA3 for the TMTP3 joint. Axes show flexion/extension (FE), abduction/adduction (ABAD) and long-axis rotation (LAR) angles in degrees at 10-degree resolution.

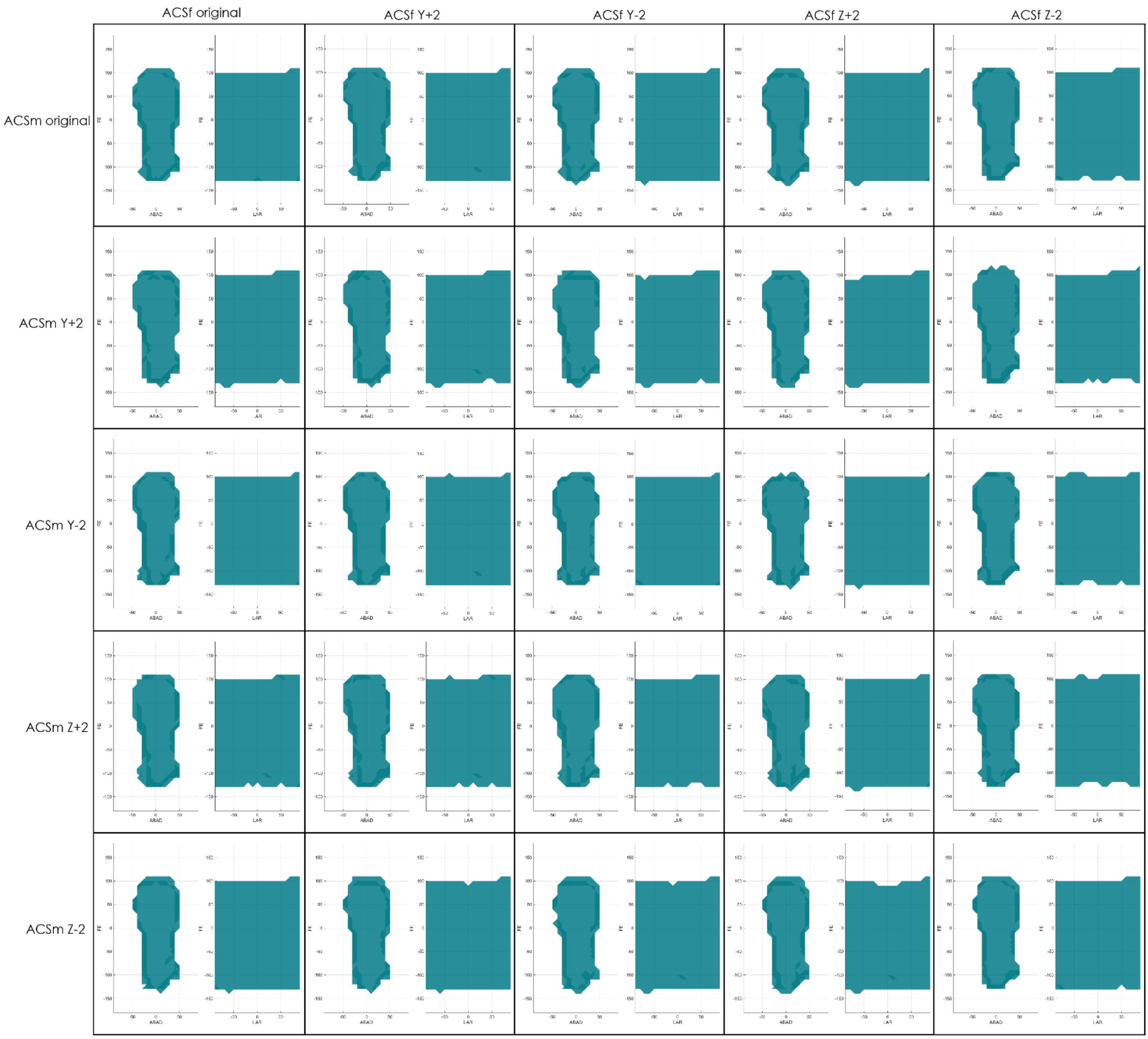

Supplementary fig.15: 2D range of motion maps for each trial of SA3 for the TMTP3 joint to show maximum rotational angles. Axes show flexion/extension (FE), abduction/adduction (ABAD) and long-axis rotation (LAR) angles in degrees at 10-degree resolution.

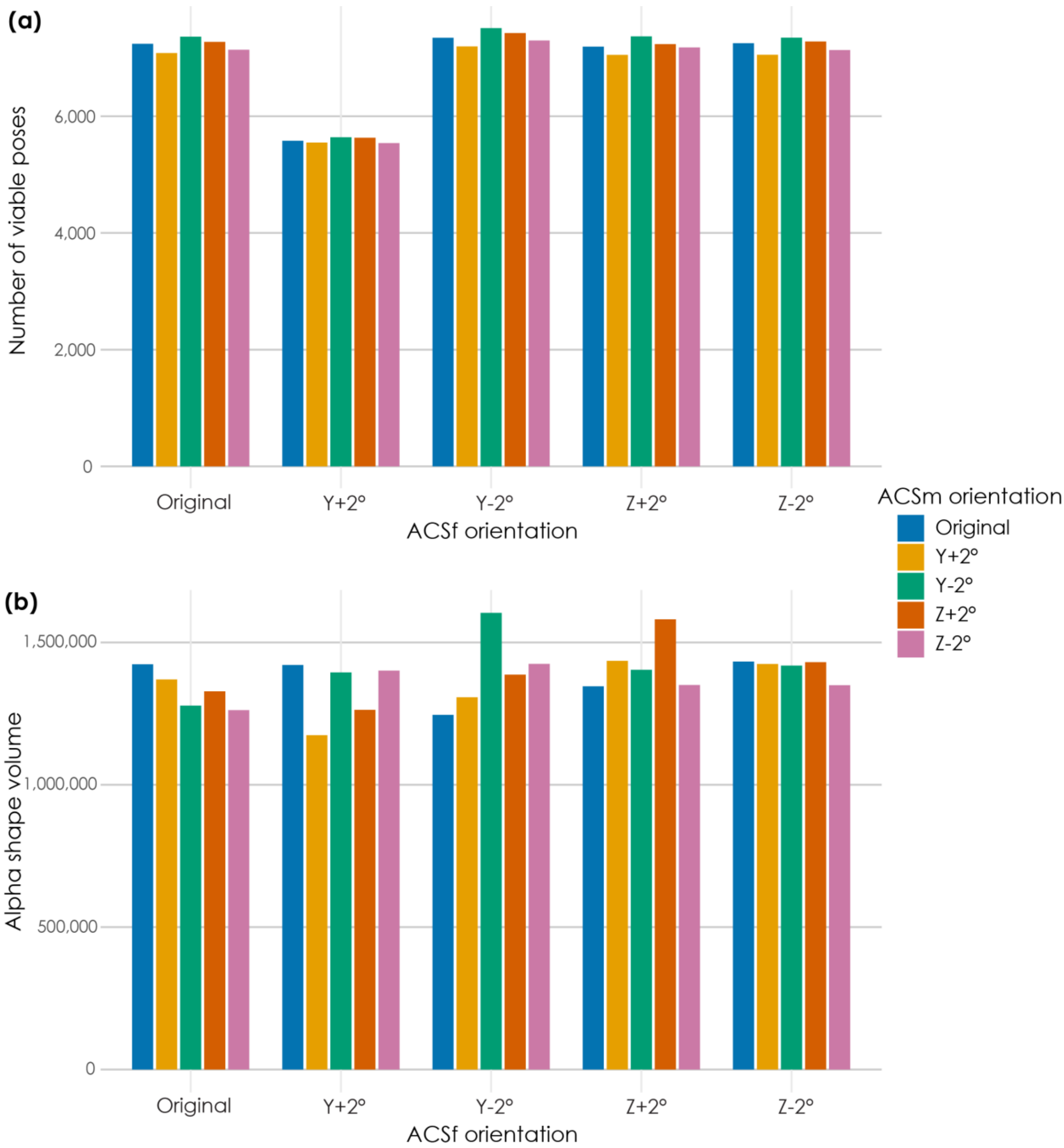

Supplementary fig.16: Number of viable poses (a) and alpha shape volume (b) for each trial of SA3 for the ankle joint.

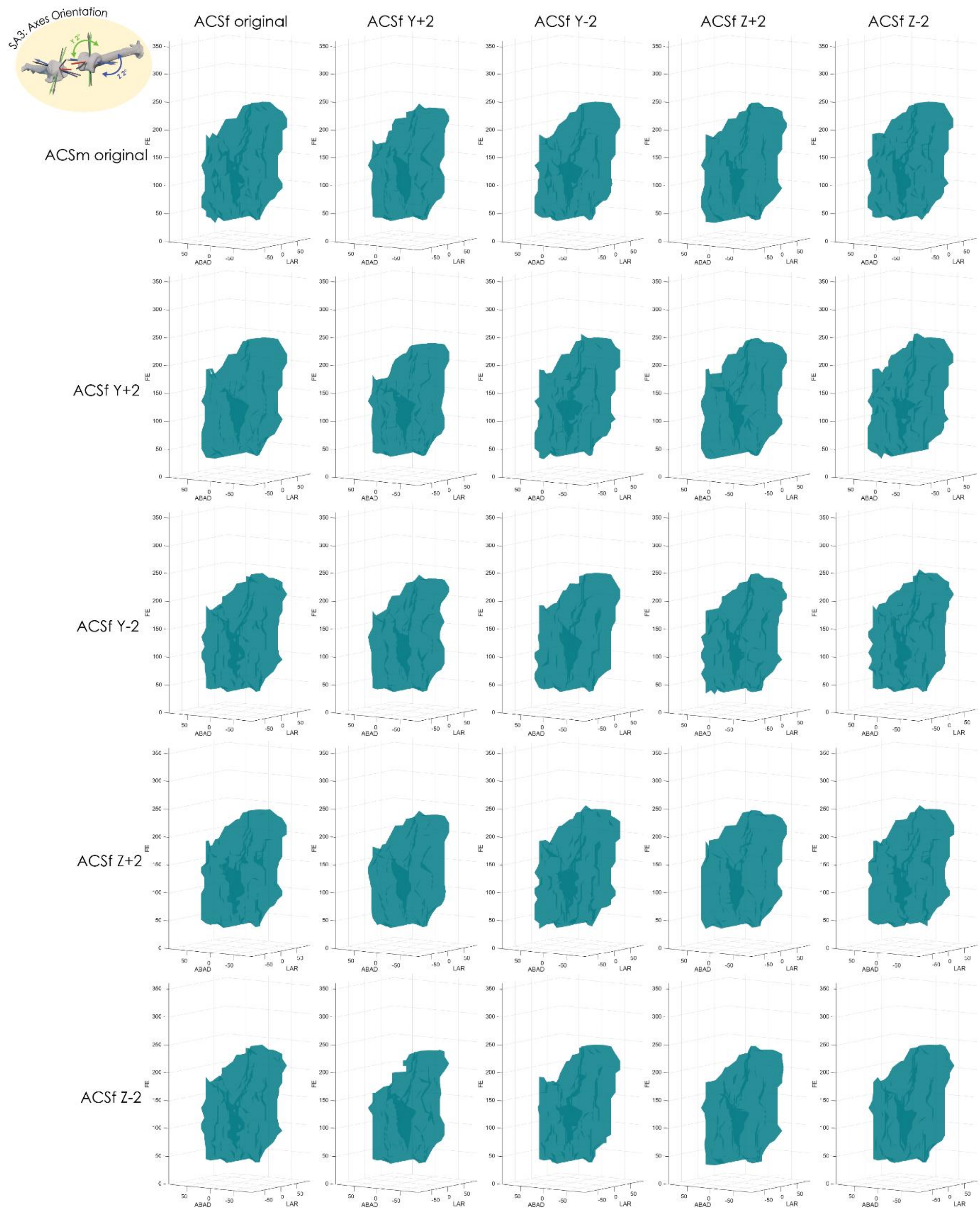

Supplementary fig.17: Cosine-corrected 3D range of motion maps for each trial of SA3 for the ankle joint. Axes show flexion/extension (FE), abduction/adduction (ABAD) and long-axis rotation (LAR) angles in degrees at 10-degree resolution.

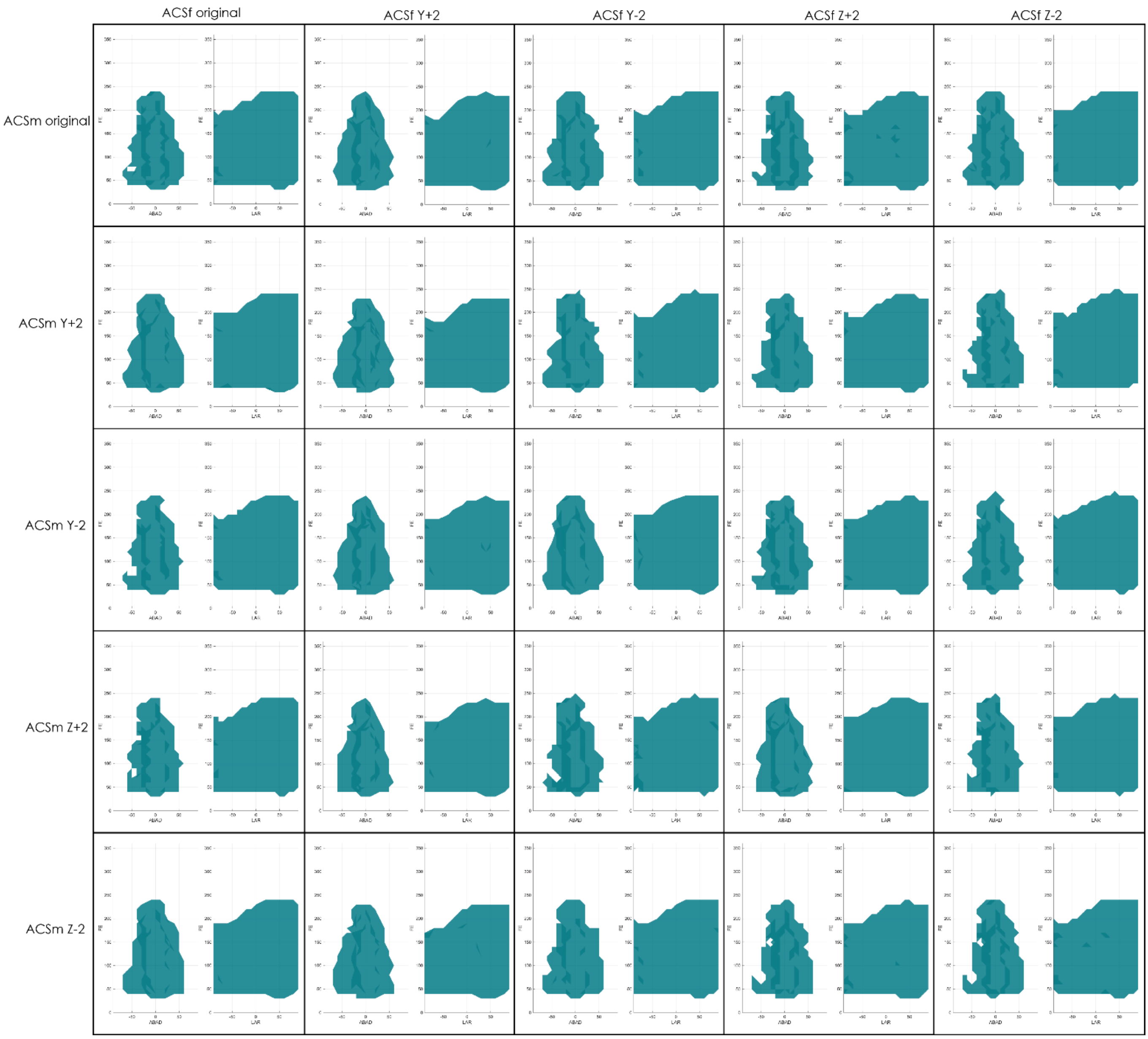

Supplementary fig.18: 2D range of motion maps for each trial of SA3 for the ankle joint to show maximum rotational angles. Axes show flexion/extension (FE), abduction/adduction (ABAD) and long-axis rotation (LAR) angles in degrees at 10-degree resolution.

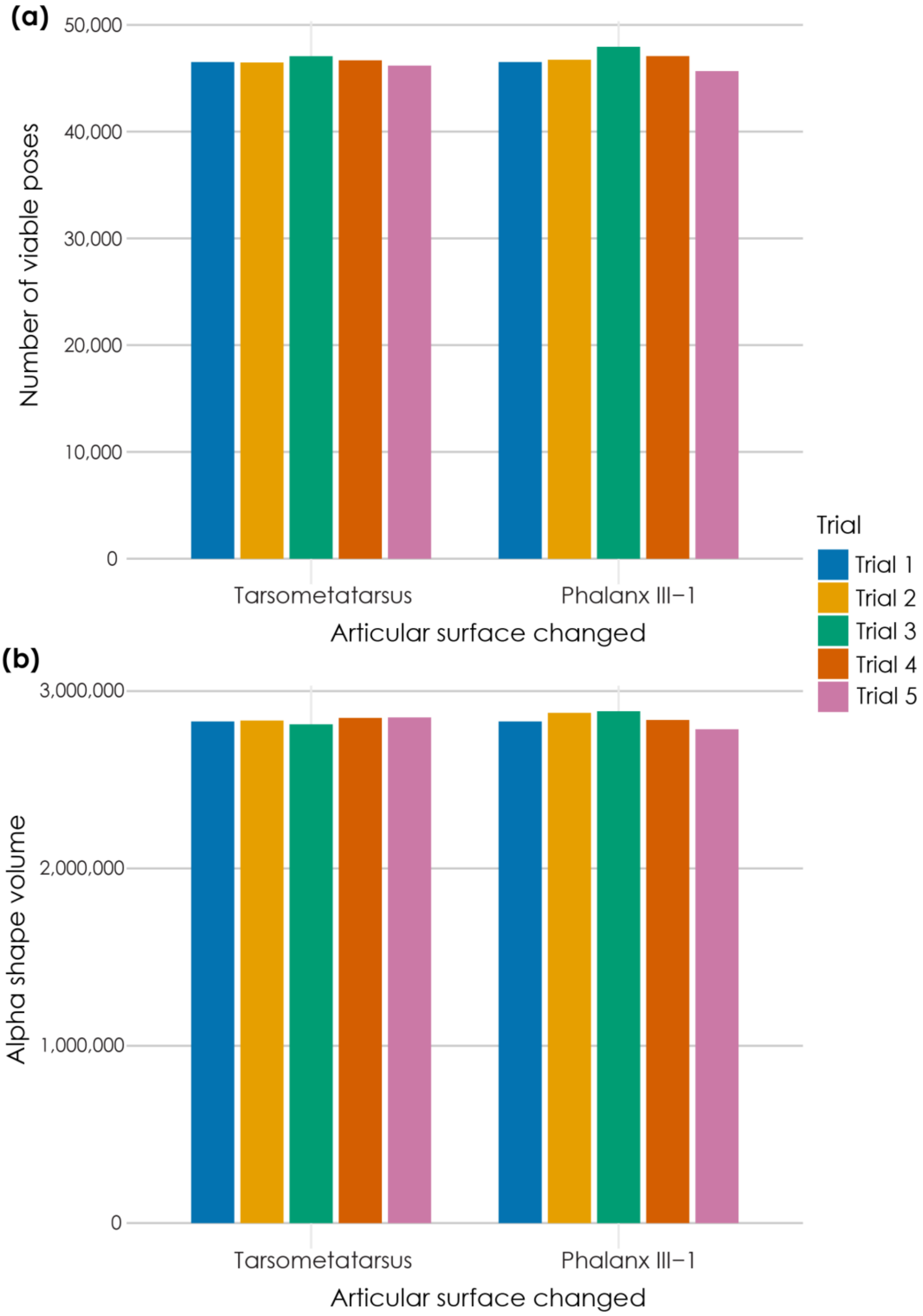

Supplementary fig.19: Number of viable poses (a) and alpha shape volume (b) for each trial of SA4 for the TMTP3 joint.

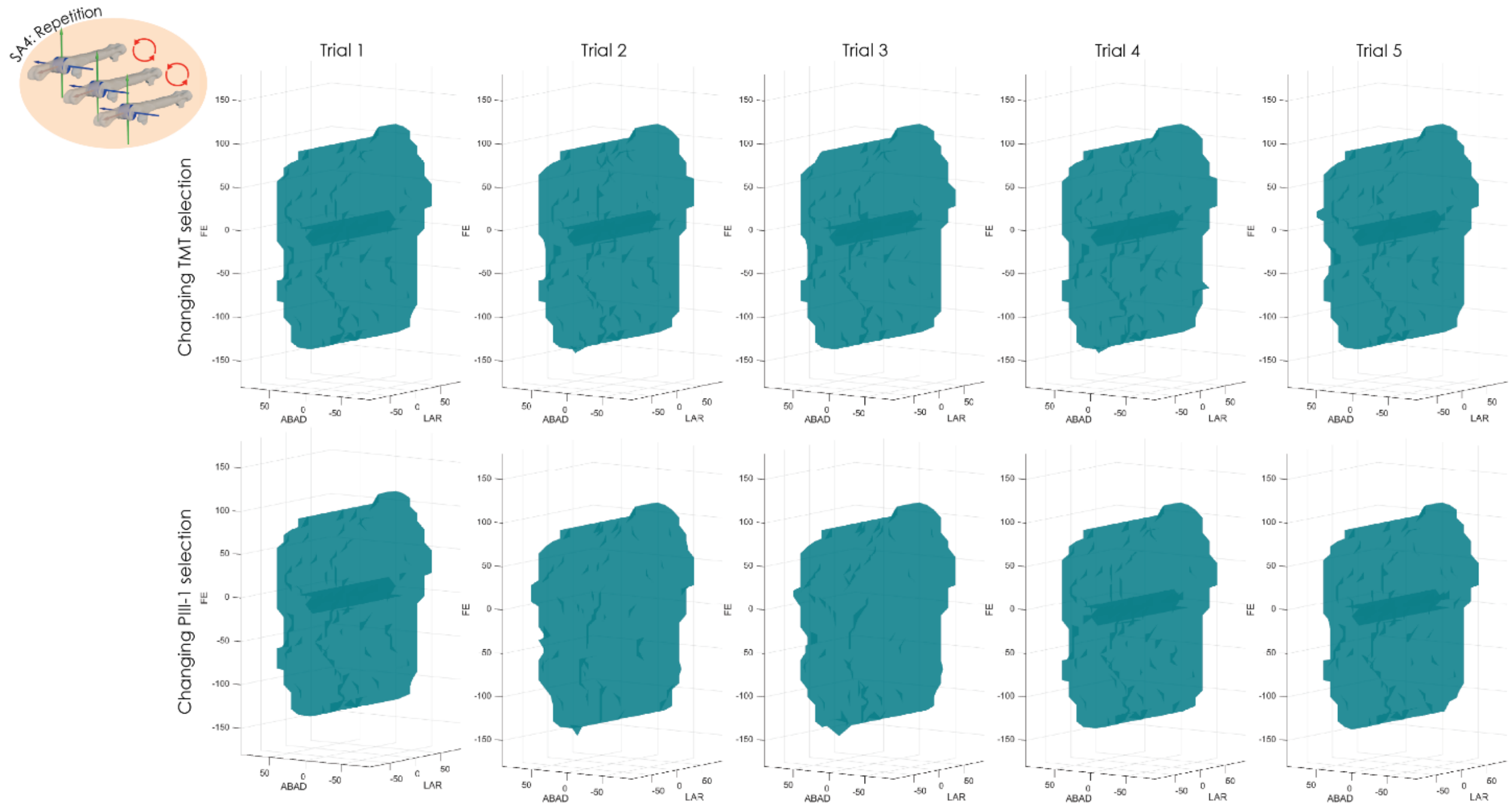

Supplementary fig.20: Cosine-corrected 3D range of motion maps for each trial of SA4 for the TMTP3 joint. Axes show flexion/extension (FE), abduction/adduction (ABAD) and long-axis rotation (LAR) angles in degrees at 10-degree resolution. Abbreviations: TMT, tarsometatarsus; PIII-1, phalanx III-1.

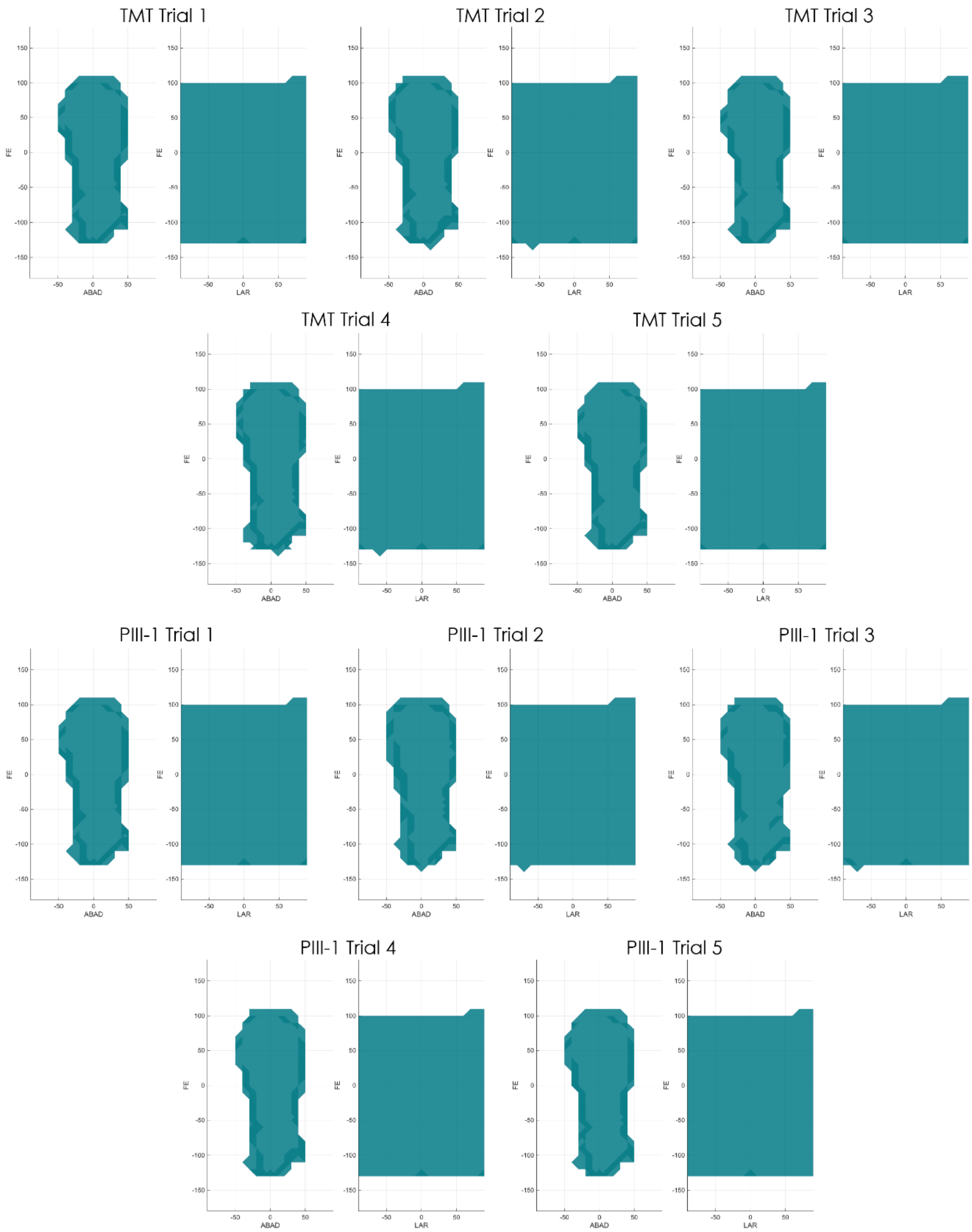

Supplementary fig.21: 2D range of motion maps for each trial of SA4 for the TMTP3 joint to show maximum rotational angles. Axes show flexion/extension (FE), abduction/adduction (ABAD) and long-axis rotation (LAR) angles in degrees at 10-degree resolution. Abbreviations: TMT, tarsometatarsus; PIII-1, phalanx III-1.

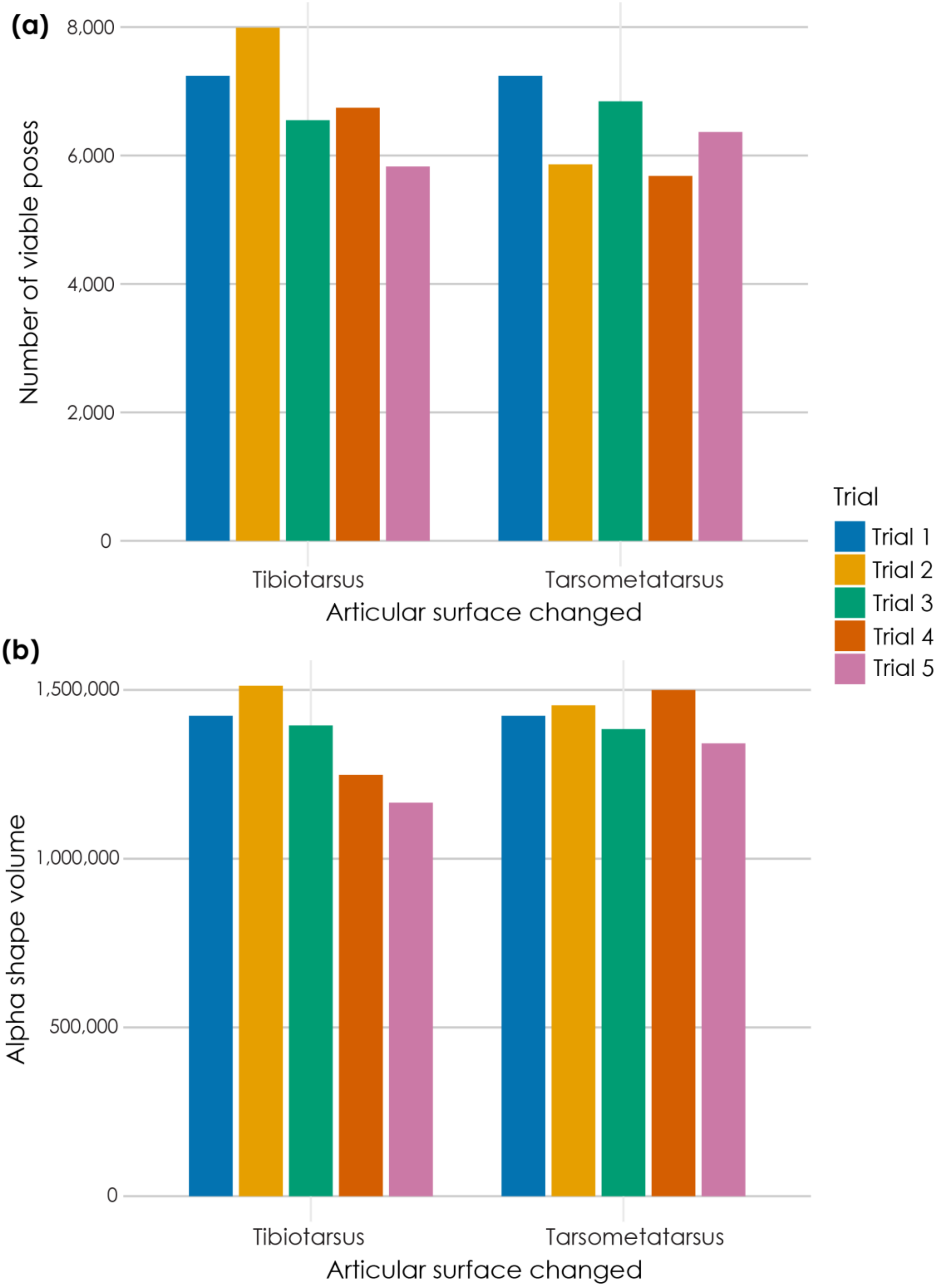

Supplementary fig.22: Number of viable poses (a) and alpha shape volume (b) for each trial of SA4 for the ankle joint.

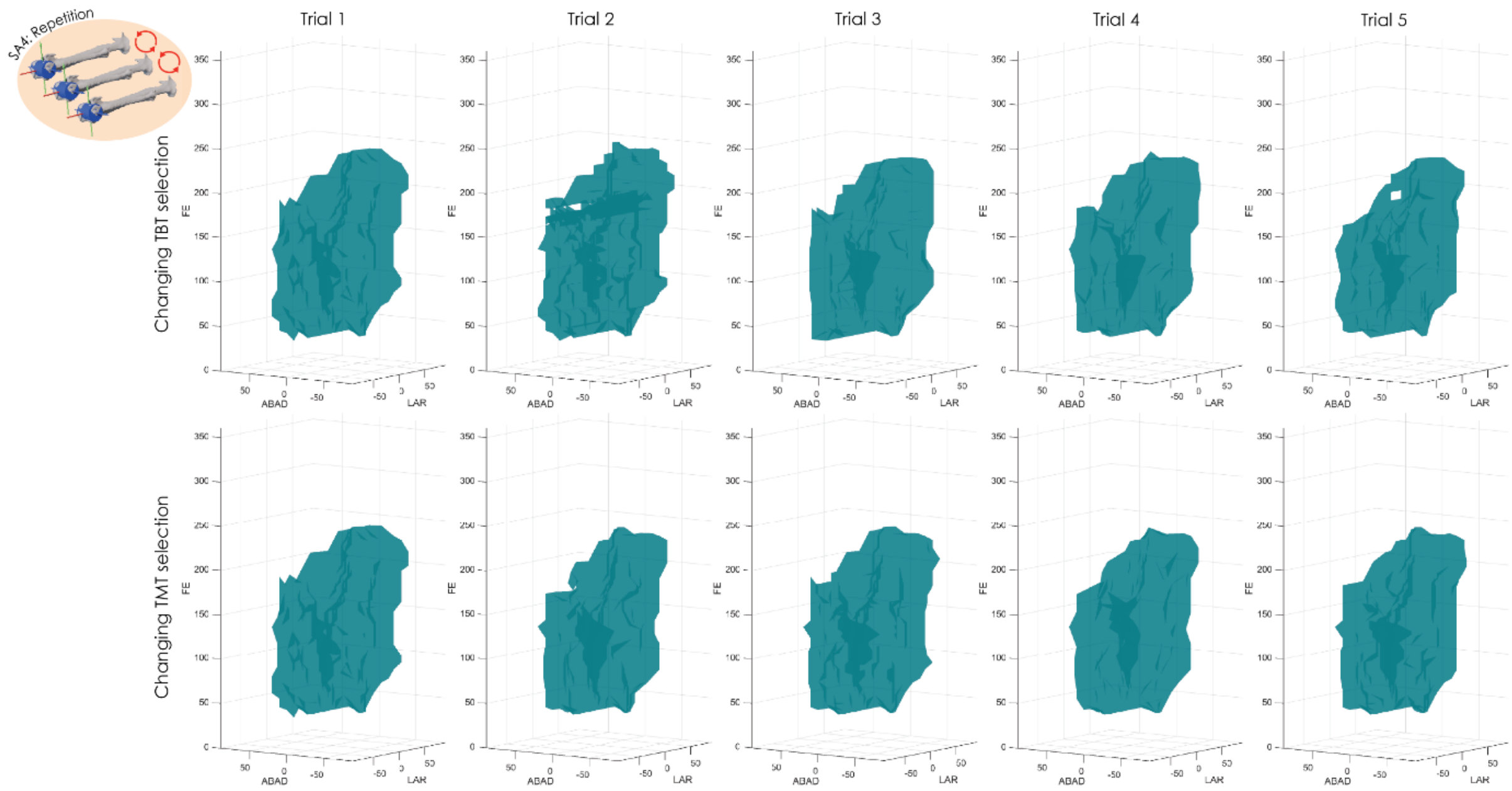

Supplementary fig.23: Cosine-corrected 3D range of motion maps for each trial of SA4 for the ankle joint. Axes show flexion/extension (FE), abduction/adduction (ABAD) and long-axis rotation (LAR) angles in degrees at 10-degree resolution. Abbreviations: TBT, tibiotarsus; TMT, tarsometatarsus.

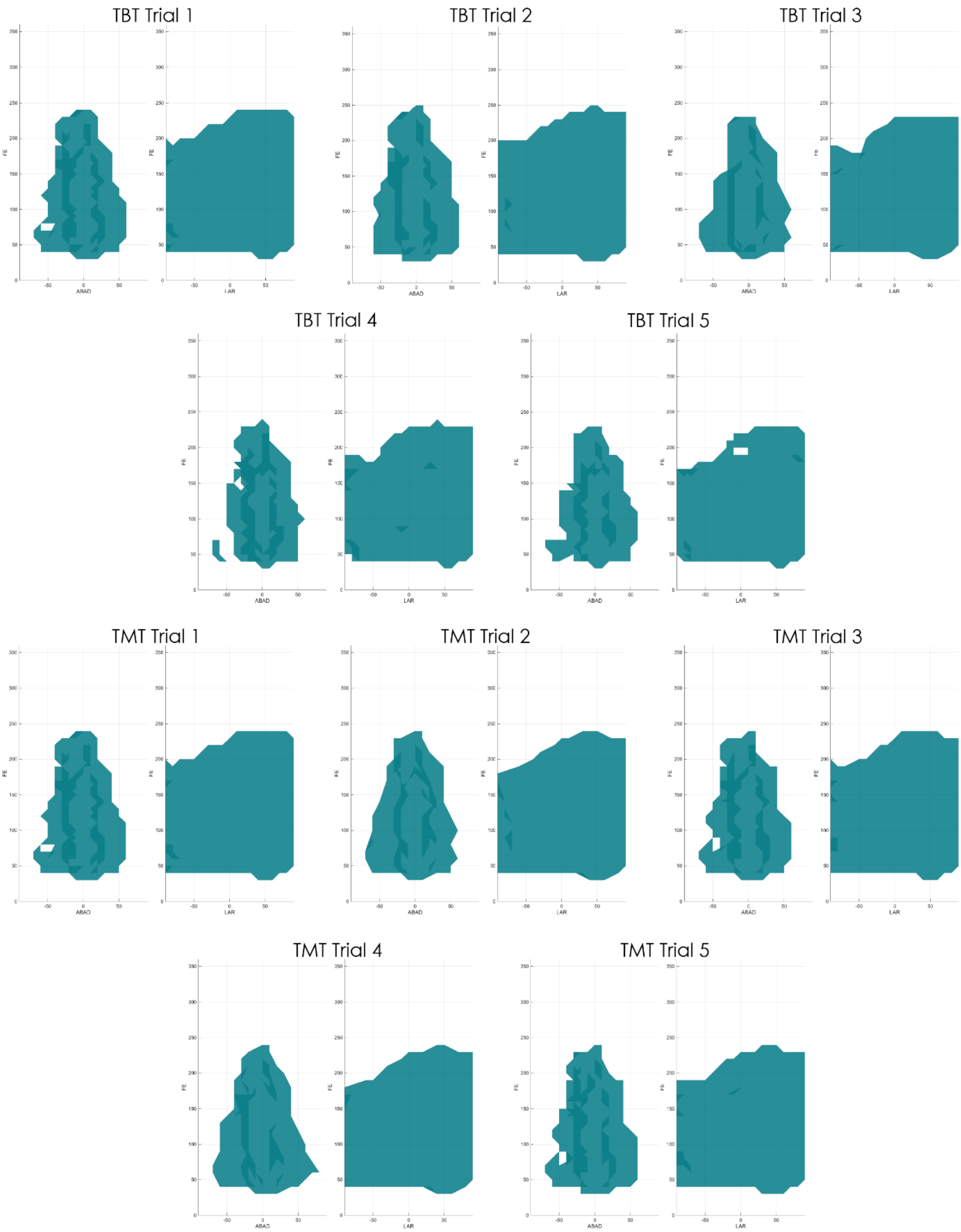

Supplementary fig.24: 2D range of motion maps for each trial of SA4 for the ankle joint to show maximum rotational angles. Axes show flexion/extension (FE), abduction/adduction (ABAD) and long-axis rotation (LAR) angles in degrees at 10-degree resolution. Abbreviations: TBT, tibiotarsus; TMT, tarsometatarsus.

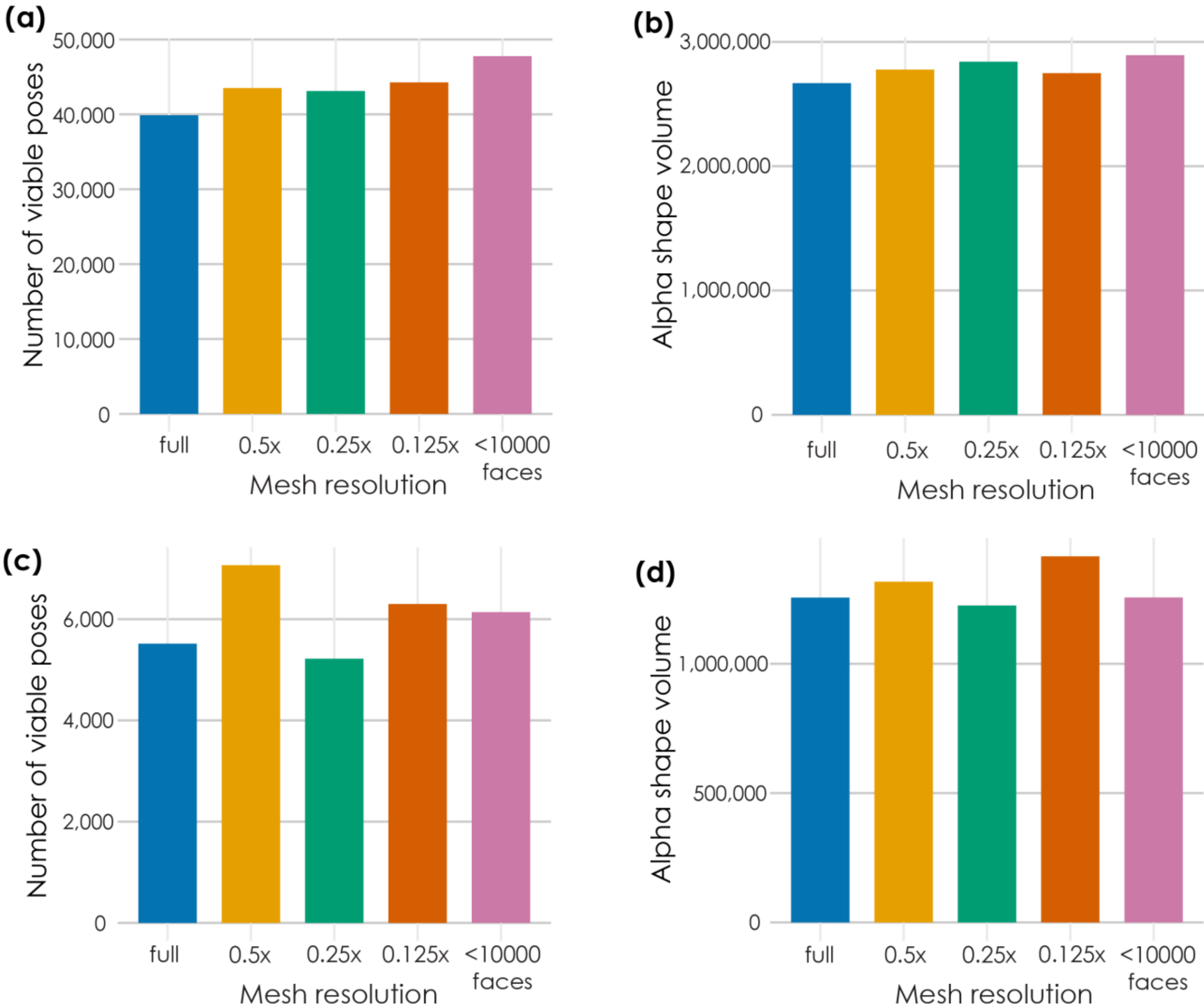

Supplementary fig.25: Number of viable poses (a) and alpha shape volume (b) for each trial of SA5 for the TMTP3 joint. Number of viable poses (c) and alpha shape volume (d) for each trial of SA5 for the ankle joint.

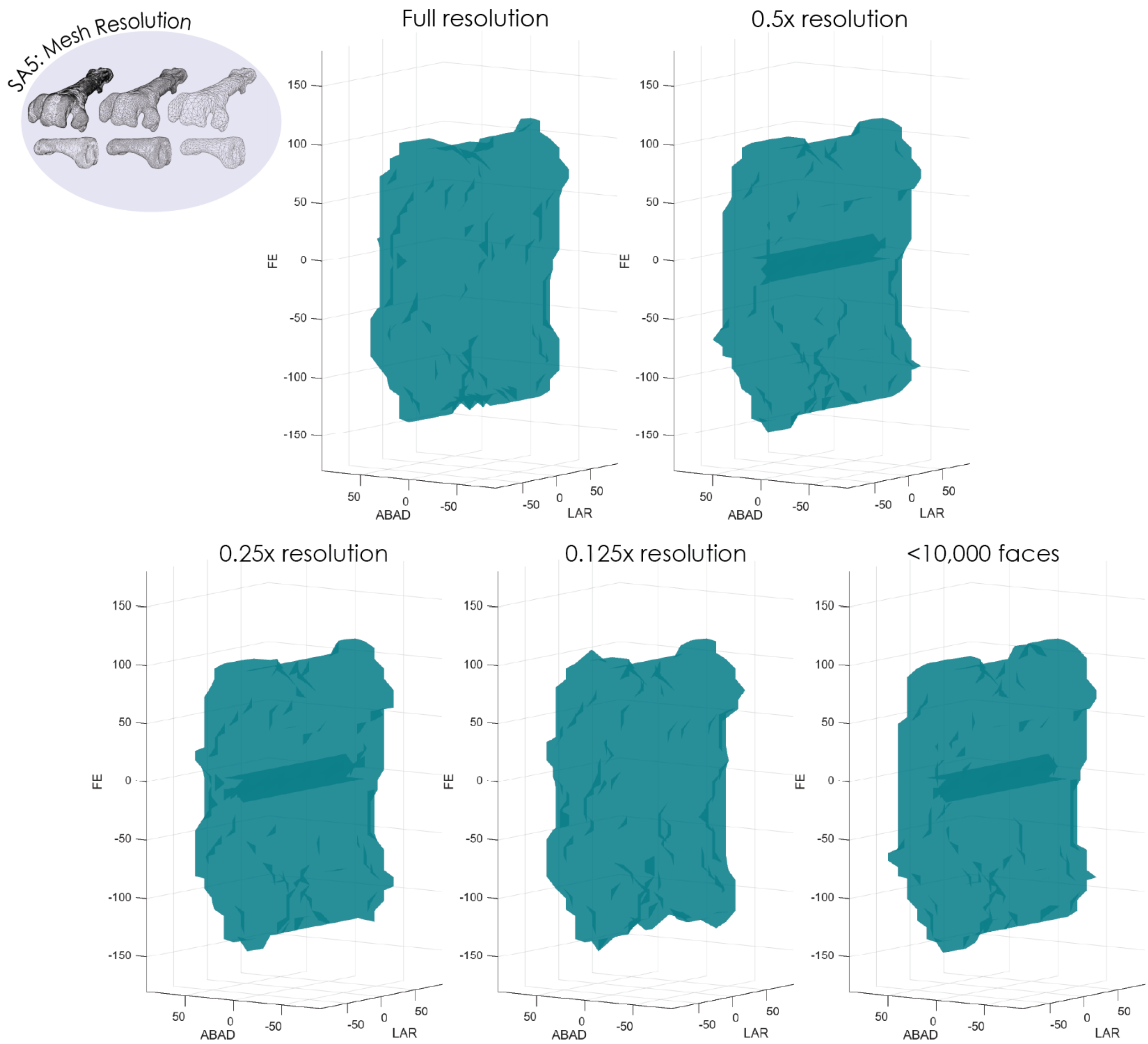

Supplementary fig.26: Cosine-corrected 3D range of motion maps for each trial of SA5 for the TMTP3 joint. Axes show flexion/extension (FE), abduction/adduction (ABAD) and long-axis rotation (LAR) angles in degrees at 10-degree resolution.

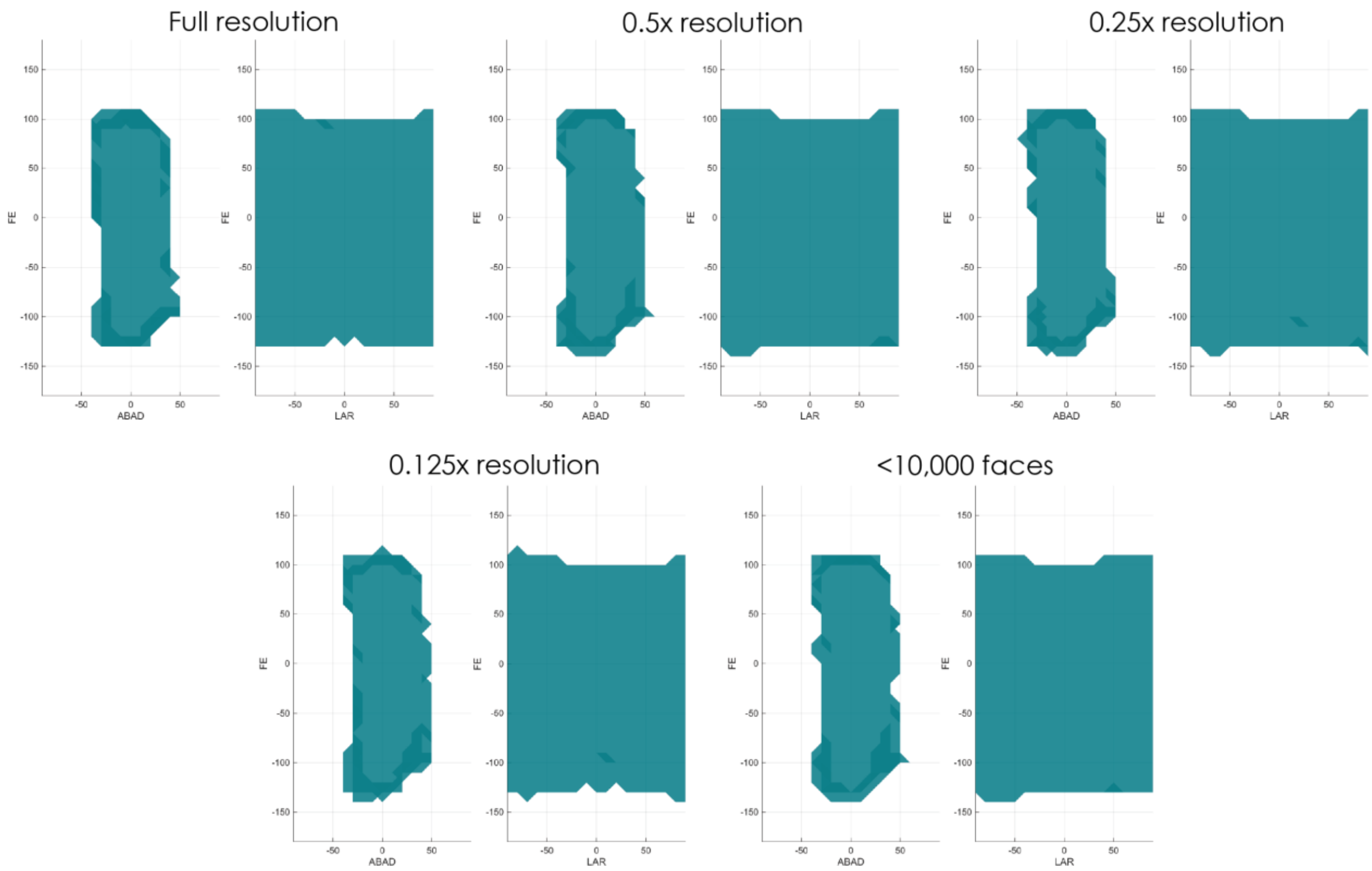

Supplementary fig.27: 2D range of motion maps for each trial of SA5 for the TMTP3 joint to show maximum rotational angles. Axes show flexion/extension (FE), abduction/adduction (ABAD) and long-axis rotation (LAR) angles in degrees at 10-degree resolution.

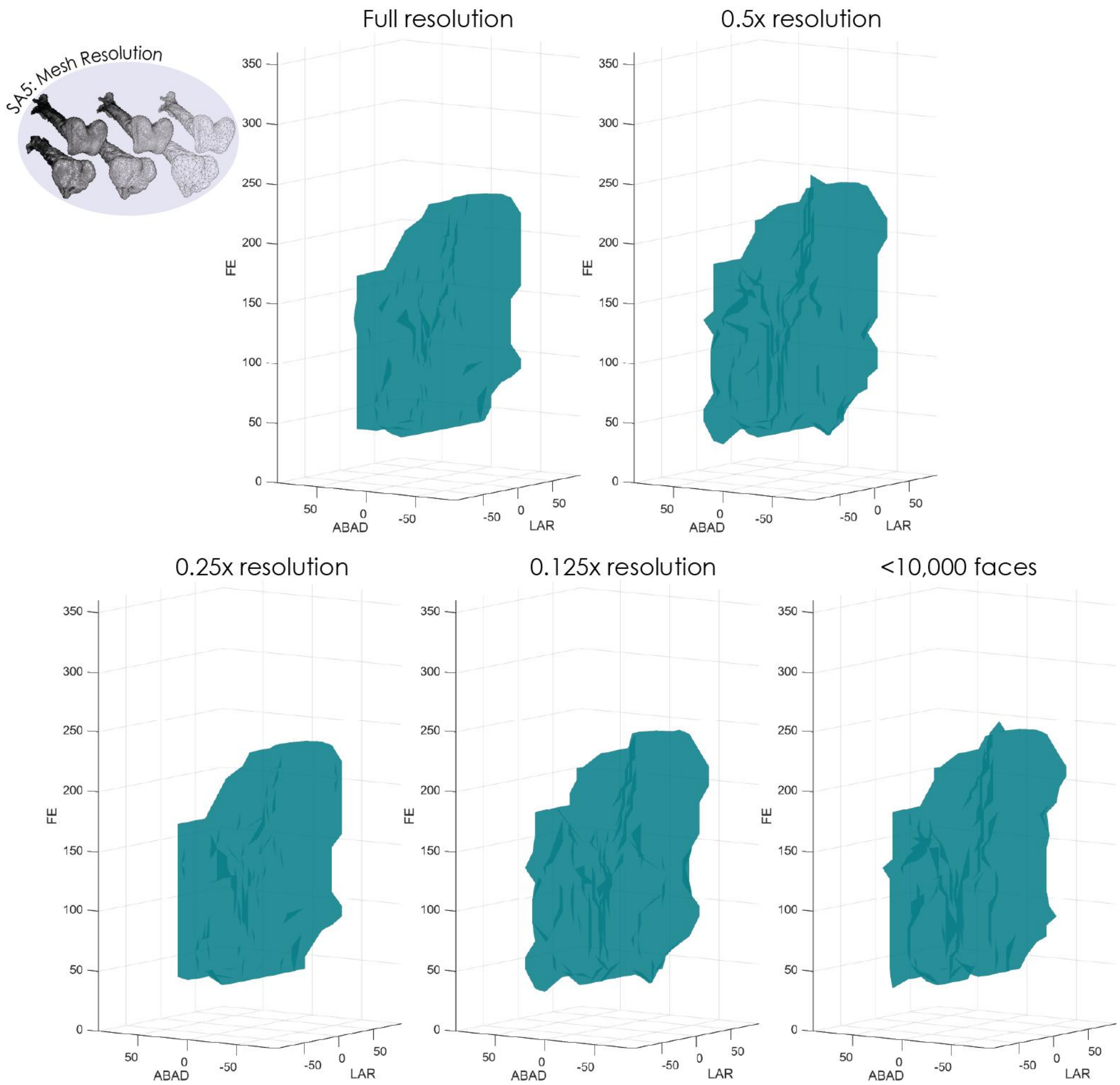

Supplementary fig.28: Cosine-corrected 3D range of motion maps for each trial of SA5 for the ankle joint. Axes show flexion/extension (FE), abduction/adduction (ABAD) and long-axis rotation (LAR) angles in degrees at 10-degree resolution.

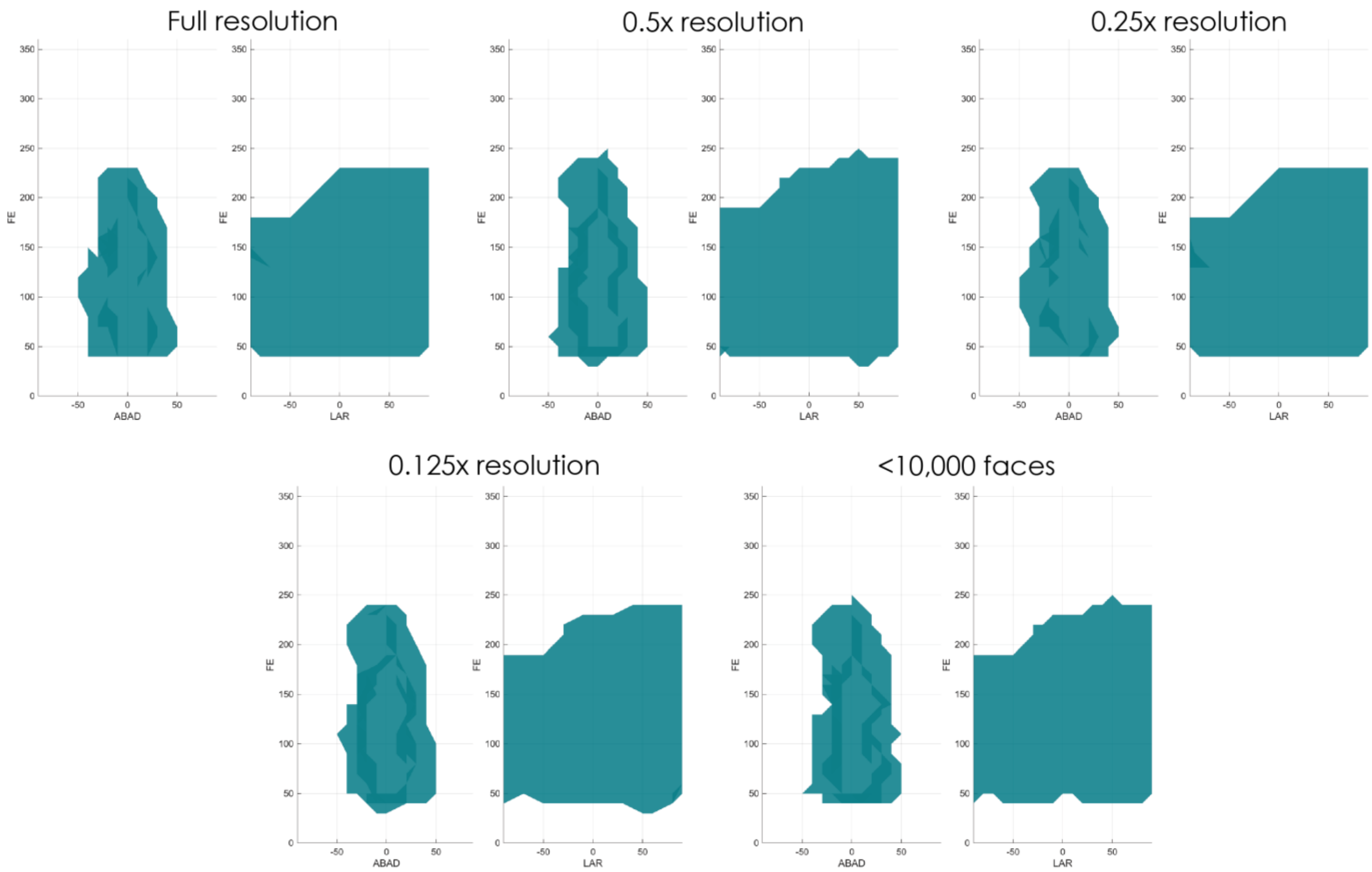

Supplementary fig.29: 2D range of motion maps for each trial of SA1 for the ankle joint to show maximum rotational angles. Axes show flexion/extension (FE), abduction/adduction (ABAD) and long-axis rotation (LAR) angles in degrees at 10-degree resolution.
